# Experimental evidence of phase transition of silica polymorphs in basaltic eucrites: implications for thermal history of protoplanetary crust

**DOI:** 10.1038/s41598-024-77544-x

**Published:** 2024-11-02

**Authors:** Rei Kanemaru, Naoya Imae, Akira Yamaguchi, Aiko Nakato, Junko Isa, Makoto Kimura, Hirotsugu Nishido, Tomohiro Usui, Takashi Mikouchi

**Affiliations:** 1 Institute of Space and Astronautical Science (ISAS), Japan Aerospace Exploration Agency (JAXA), Sagamihara, Kanagawa 252-5210 Japan; 2 National Institute of Polar Research (NIPR), Tachikawa, Tokyo 190-8518 Japan; 3 The Graduate University for Advanced Studies (SOKENDAI), Hayama, Kanagawa 240-0115 Japan; 4 Earth-Life Science Institute (ELSI), Tokyo Institute of Technology, Ookayama, Tokyo 152-8550 Japan; 5 Cold Pine Observatory, Chigasaki, Kanagawa 253-0025 Japan; 6 Okayama University of Science (OUS), Okayama, Okayama 700-0005 Japan; 7 The University Museum, The University of Tokyo, Bunkyo-Ku, Tokyo, 113-0033 Japan

**Keywords:** Eucrite, Silica polymorphs, Isothermal experiments, Thermal metamorphism, Cathodoluminescence, Planetary science, Solid Earth sciences

## Abstract

Silica polymorphs occur under various pressures and temperature conditions, and their characteristics can be used to better understand the complex metamorphic history of planetary materials. Here, we conducted isothermal heating experiments of silica polymorphs in basaltic eucrites to assess their formation and stability. We revealed that each silica polymorph exhibits different metamorphic responses: (1) Quartz recrystallizes into cristobalite when heated at ≥ 1040 °C. (2) Monoclinic (MC) tridymite recrystallizes into no other polymorphs when heated at ≤ 1070 °C. (3) Silica glass recrystallizes into quartz when heated at 900–1010 °C, and recrystallize into cristobalite when heated at ≥ 1040 °C. These results suggest that MC tridymite in eucrites does not recrystallize into other polymorphs during the reheating events, nor does it recrystallize from other silica phases below the solidus temperature of eucrite (~ 1060 °C). Additionally, we found that pseudo-orthorhombic (PO) tridymite crystallizes from quenched melts in the samples heated at ≥ 1070 °C. Previously, cristobalite has been considered as the initial silica phase, which crystallizes from eucritic magma. Our findings suggest that the first crystallizing silica minerals may not always be cristobalite. These require a reconsideration of the formation process of silica minerals in eucrites.

## Introduction

Basaltic eucrites originate from the surface crustal material of a protoplanet 4 Vesta in the early Solar System^1^. Eucrites are primarily composed of pyroxene, plagioclase, and several accessory minerals such as silica minerals, troilite, ilmenite, chromite, and phosphate. When subjected to extensive thermal and shock metamorphism, eucrites undergo significant alterations in both petrographic and chemical features^2–4^. Understanding these metamorphic processes is crucial for deciphering the formation and evolutionary history of eucrites.

Silica mineral has several polymorphs under different pressure and temperature conditions^5,6^. The characterization of silica polymorphs provides information about the thermal and shock history of parent bodies^7,8^. Recently, a formation model for silica minerals in eucrites has been proposed. It suggests that the variations in silica polymorphs within these meteorites are related to cooling rates, as determined by the mineralogical features of pyroxene^9^. Hereafter, we refer to this model as the “Ono model”. This model argues that cristobalite is the first silica polymorph to crystallize from eucritic magma, and subsequently recrystallize into quartz and tridymite during the cooling process. Additionally, the model proposed that tridymite recrystallized into quartz as a result of secondary heating events. This hypothesis is supported by their isothermal heating experiments on a cumulate eucrite Yamato 980433 (Y 980433), showing that monoclinic (MC) tridymite recrystallized into quartz when heated at 800 °C for 96 h^9^. However, our preliminary observations on Y 980433 revealed two potential issues with their experiments that may affect the parts of the Ono model:

1. Y 980433 suffered from strong shock metamorphism (shock degree D^3^); and many of the MC tridymite converted into silica glass^10^.
2. Quartz was originally present in Y 980433 before heating^11^.

Consequently, it is unclear whether the recrystallization from MC tridymite into quartz occurred, since they did not observe the same areas before and after heating.

In this study, we performed a mineralogical study on silica minerals in experimentally heated basaltic eucrites to better understand the effects of thermal metamorphism on the parent body. Specifically, to address the above mentioned two potential issues, in several samples, we observed the same areas before and after heating to clarify phase transitions of the silica minerals. We utilized the cathodoluminescence (CL) imaging technique to characterize silica polymorphs in detail. The CL color reflects the physical and chemical state of the mineral, making it an invaluable tool for differentiating among silica polymorphs^12^.

## Results

### Overall sample textures of before and after the heating

Three basaltic eucrites for our study: of Asuka-87272 (A-87272) (subsamples, 79 and, 88), Agoult, and Hammada al Hamra 262 (HaH 262) were selected for the heating experiments (Tables 1 and 2). We also used the chips and run products of HaH 262 from a previous heating experiment^4^. These starting materials differ in their petrologic types and shock degrees (Table 1)^2,3,13^. The detailed petrologic features for unheated samples are summarized in Supplementary Figs. 1, 2 and 3. The solidus temperature of basaltic eucrite is ~ 1060 °C^14^, around which the effect of thermal metamorphism becomes significantly pronounced, leading to the formation of small amounts of partial melts. These melts were detected in samples heated at ≥ 1040 °C (Supplementary Figs. 4, 5, 6and 7), filling fractures in minerals, especially in plagioclase. We also observed secondary phases such as rounded pyroxene and tiny plagioclase aggregates. The partial melts are especially abundant in A-87272, but found little in Agoult.

**Table 1:** Studied basaltic eucrites and these petrographic features.

Sample	Classification	Petrologic type	Shock degree	Silica minerals				
				Quartz	Tridymite	Cristobalite	MX tridymite	Silica glass
Agoult	Recrystallized breccia	5	A	–	Yes (major)	–	–	–
A-87272	Unbrecciated	7	E	Yes (minor)	Yes (minor)	–	Yes	Yes
HaH 262	Unbrecciated	4	B	Yes (major)	–	Yes (minor)	–	–

**Table 2:** Conditions of heating experiments.

Heating temperatures and time	Samples		Vacuum conditions	Sample container	Cooling conditions
*(Experiment 1) Electric furnace@JAXA*			*Turbo-molecular pump*		
800 °C for 100 h	Agoult	A-87272, 79	1.63 × 10^–6^ Pa	Stainless board	Air quenching
900 °C for 100 h	Agoult	A-87272, 79	1.70 × 10^–5^ Pa	Stainless board	Air quenching
1000 °C for 100 h	Agoult	A-87272, 79	1.90 × 10^–5^ Pa	Stainless board	Air quenching
*(Experiment 2) Vertical furnace @NIPR*			*Rotary pump*		
1010 °C for 100 h	HaH 262		~ 10^−1^ Pa	Quartz tube	Air quenching
1040 °C for 100 h	HaH 262, Agoult	A-87272, 88	~ 10^−1^ Pa	Quartz tube	Air quenching
1070 °C for 100 h	HaH 262, Agoult	A-87272, 88	~ 10^−1^ Pa	Quartz tube	Air quenching
Heating temperatures and time	Samples		Heating conditions	Sample container	Cooling conditions
(Experiment3*) Vertical furnace @Univ. Tokyo			*1 bar gas-mixing*		
1050 °C for 24 h	HaH 262		log fO_2_ = IW-1 (gas mixture of CO_2_-H_2_)	Pt foil	Air quenching
1070 °C for 24 h	HaH 262		log fO_2_ = IW-1 (gas mixture of CO_2_-H_2_)	Pt foil	Air quenching
1100 °C for 24 h	HaH 262		log fO_2_ = IW-1 (gas mixture of CO_2_-H_2_)	Pt foil	Air quenching
1100 °C for 24 h	HaH 262		log fO_2_ = IW-1 (gas mixture of CO_2_-H_2_)	Pt foil	Cooling to 1030 °C at 10 °C/day

### Characterization of silica phases in starting materials

**Fig. 1:**
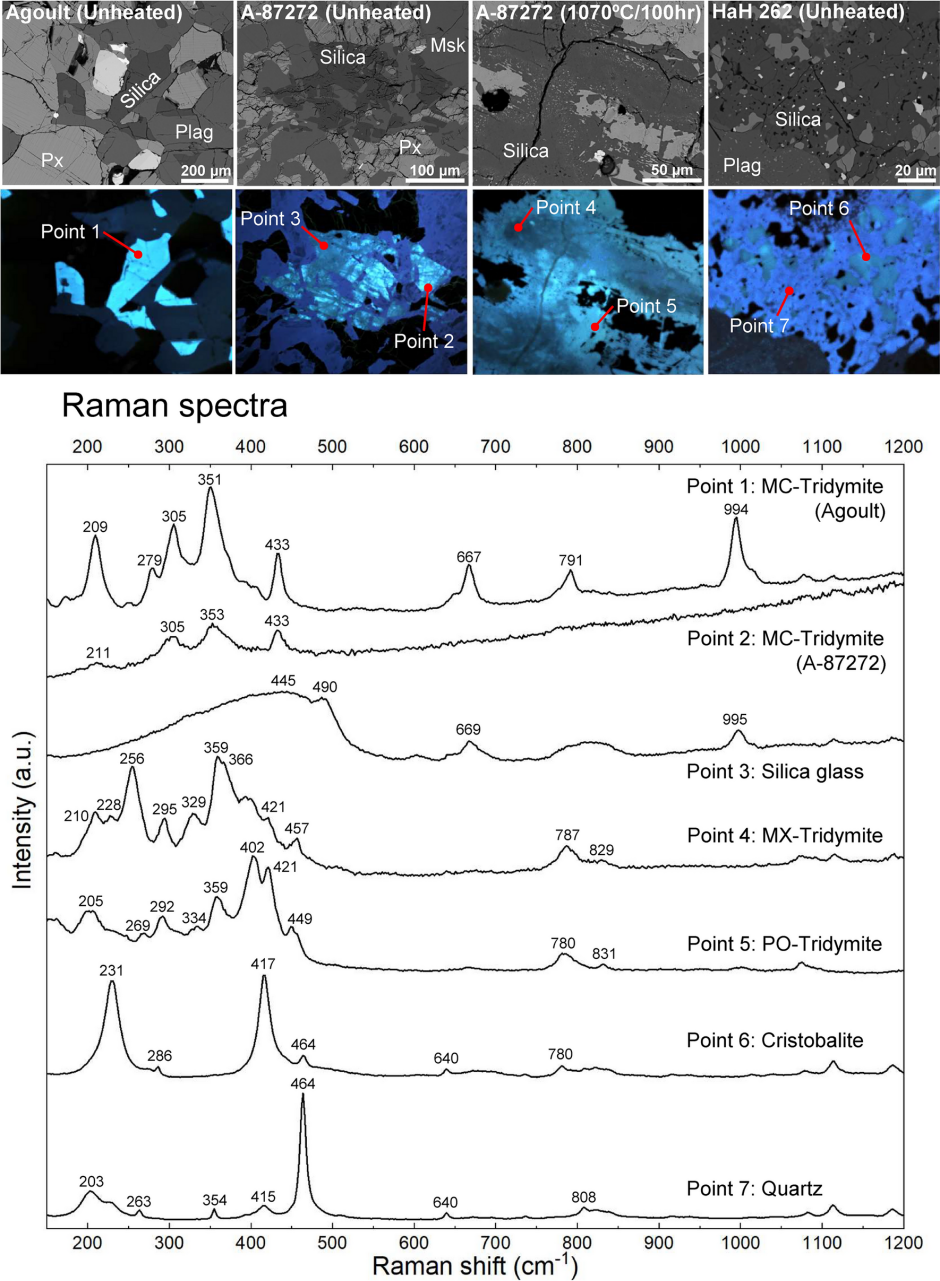
Identification of silica polymorphs in the studied eucrites using ChromaCL images and Raman spectra. Silica polymorphs can be distinguished by the variations in CL colors. Note that the CL color does not directly identify silica phases. We deliberately use relative CL colors with subtle change in their tone to distinguish silica phases. Plag = plagioclase; Px = pyroxene; Msk = maskelynite.

**Fig. 2:**
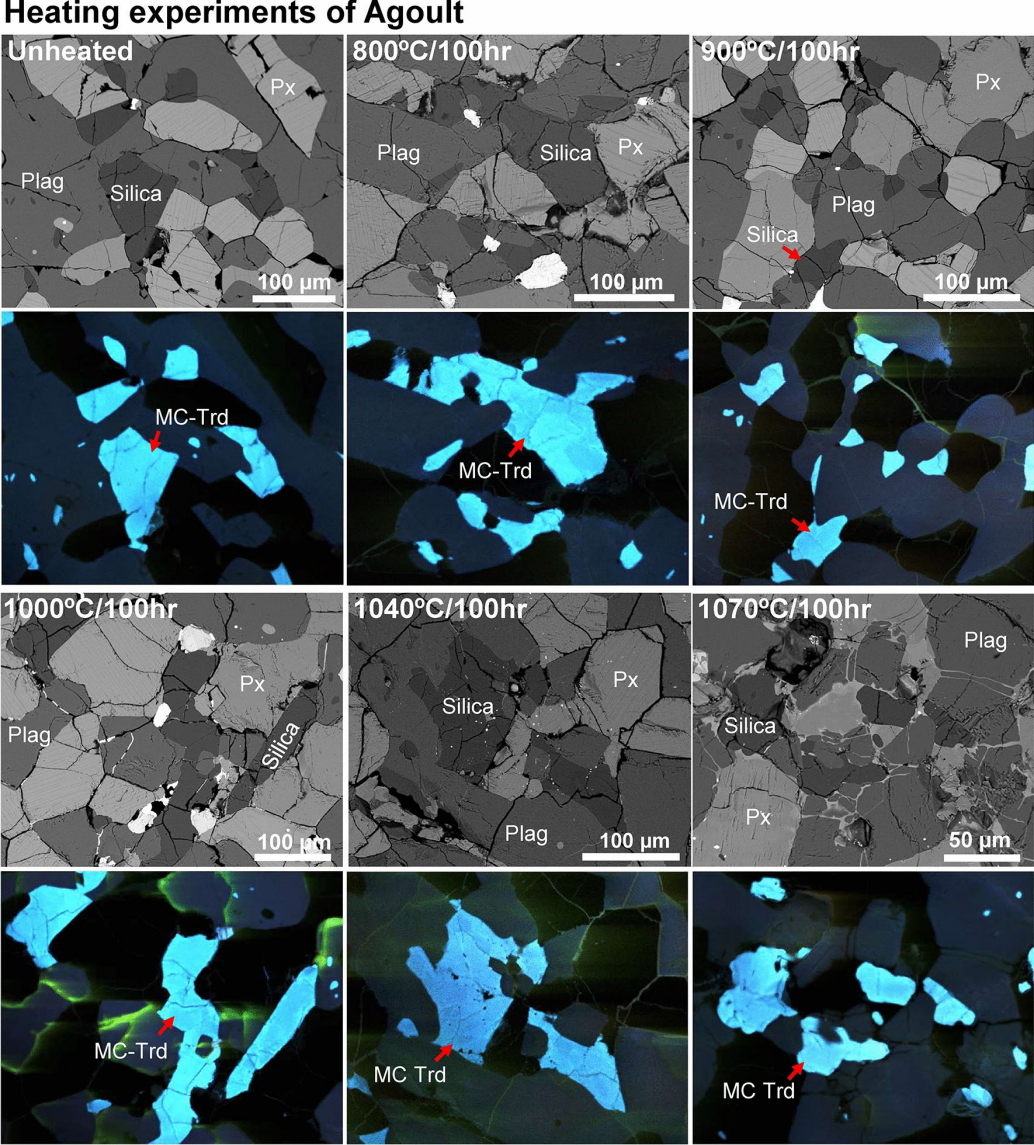
BSE and ChromaCL images of silica phases in unheated and experimentally heated Agoult (Ex. 1). All of the silica minerals in Agoult show bright aqua-blue CL color, representing MC tridymite. Agoult includes no other silica polymorphs. Plag = plagioclase; Px = pyroxene; MC-Trd = monoclinic tridymite.

**Fig. 3:**
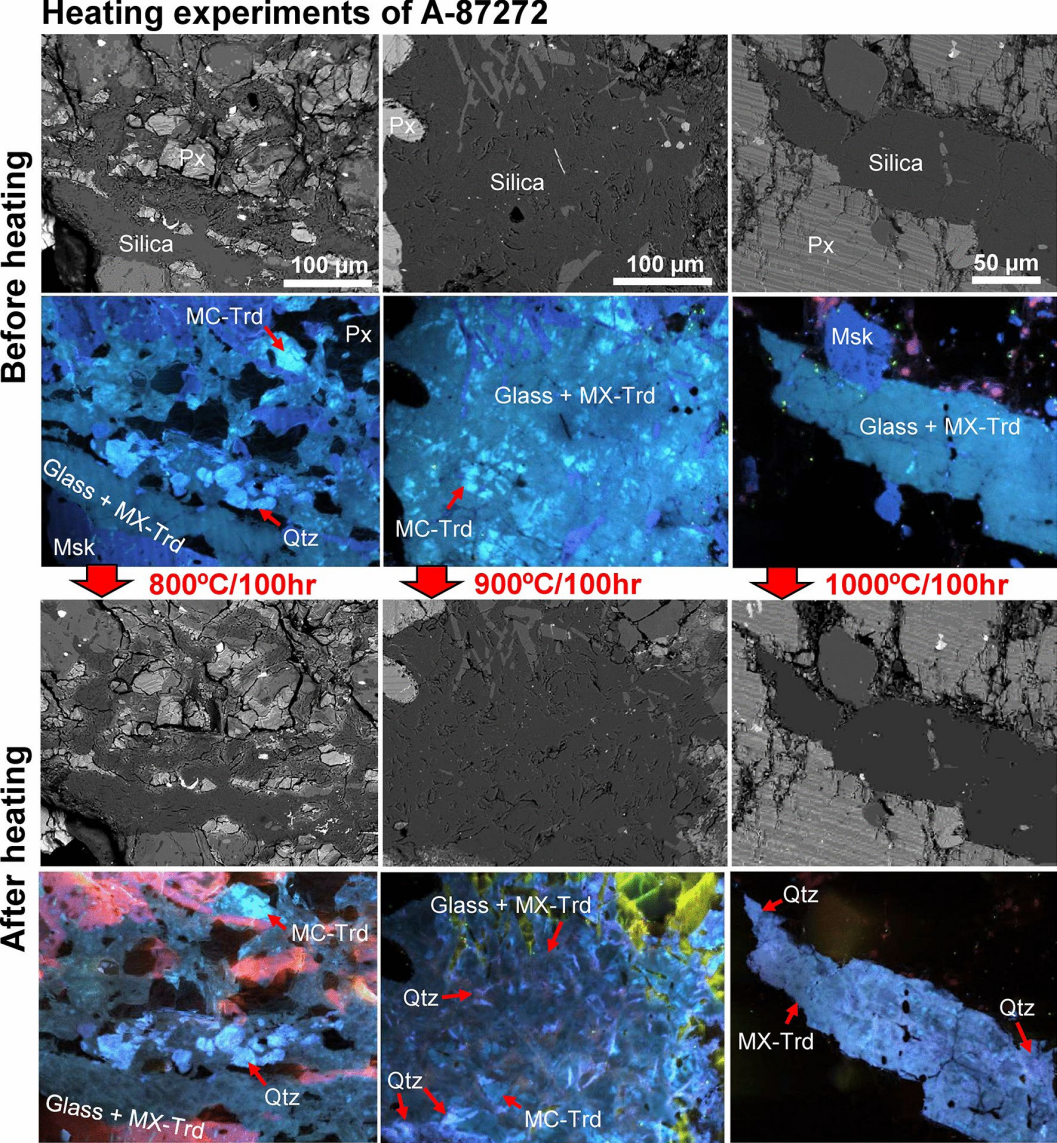
BSE and ChromaCL images of silica phases in unheated and experimentally heated A-87272 (Ex. 1 at ISAS). The top images show observations before heating, while the bottom images show the same area after heating. In the sample heated at 800 °C, there is no clear difference in the CL colors. Both MC tridymite and quartz do not recrystallize into other phases. On the other hand, in the sample heated at 900 and 1000 °C, purplish CL colors are observed on the aggregate composed by silica glass and MX tridymite. These portions are identified as quartz. Px = pyroxene; Msk = maskelynite; Qtz = quartz; MC-Trd = monoclinic tridymite; MX-Trd = monoclinic X tridymite.

**Fig. 4:**
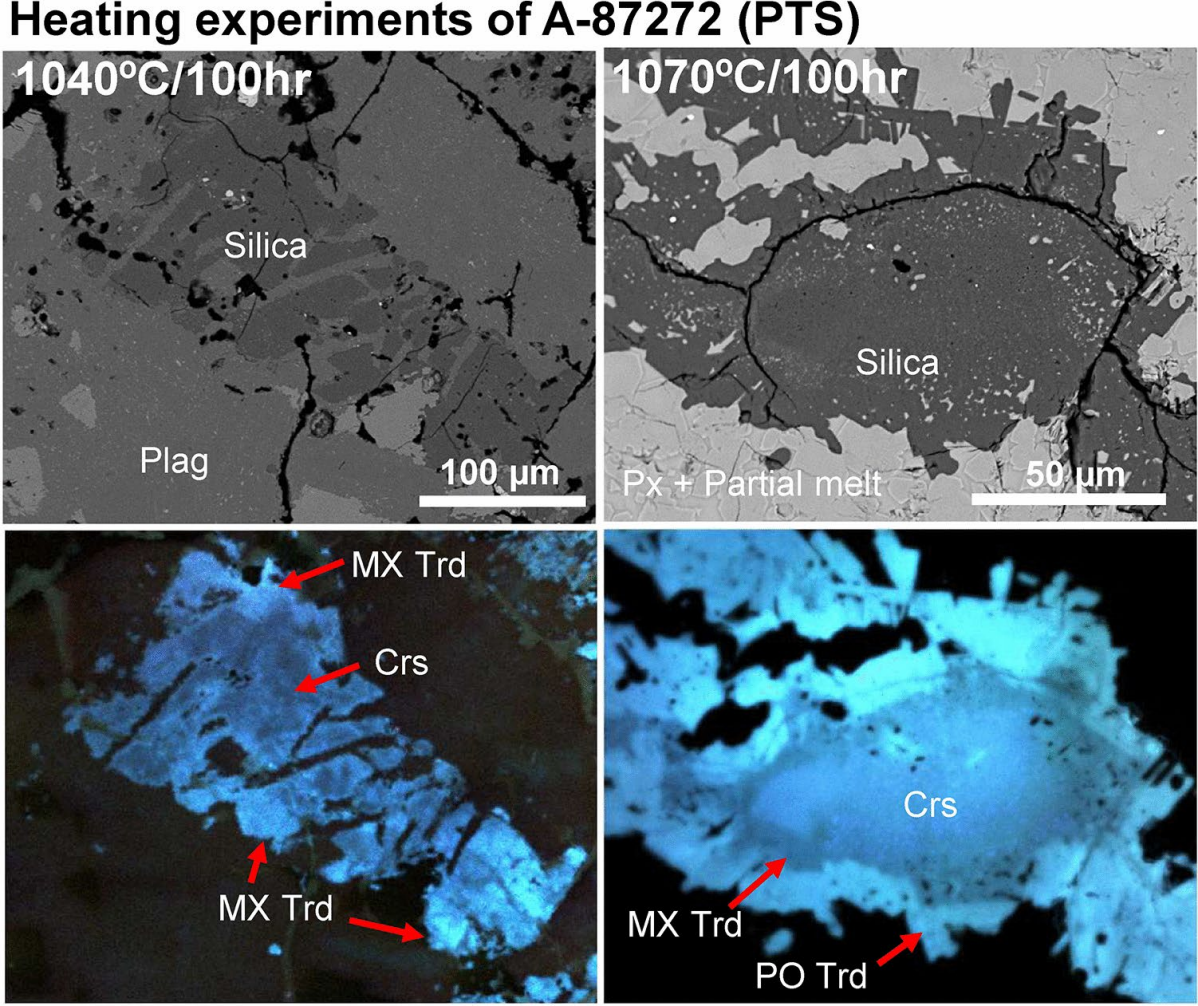
BSE and ChromaCL images of silica phases in A-87272 heated at 1040–1070 °C for 100 h (Ex. 2 at NIPR). In the A-87272 heated at 1040 °C for 100 h, the silica grains show the zoning structure with a core of cristobalite and an outer rim of MX tridymite. On the other hand, in the A-87272 heated at 1070 °C for 100 h, the silica grains show a structure with an outermost rim of PO tridymite, a core of cristobalite, and an intermediate layer of MX tridymite. Px = pyroxene; Plag = plagioclase; Qtz = quartz; Crs = cristobalite; MX-Trd = monoclinic X tridymite; PO-Trd = pseudo-orthorhombic tridymite.

**Fig. 5:**
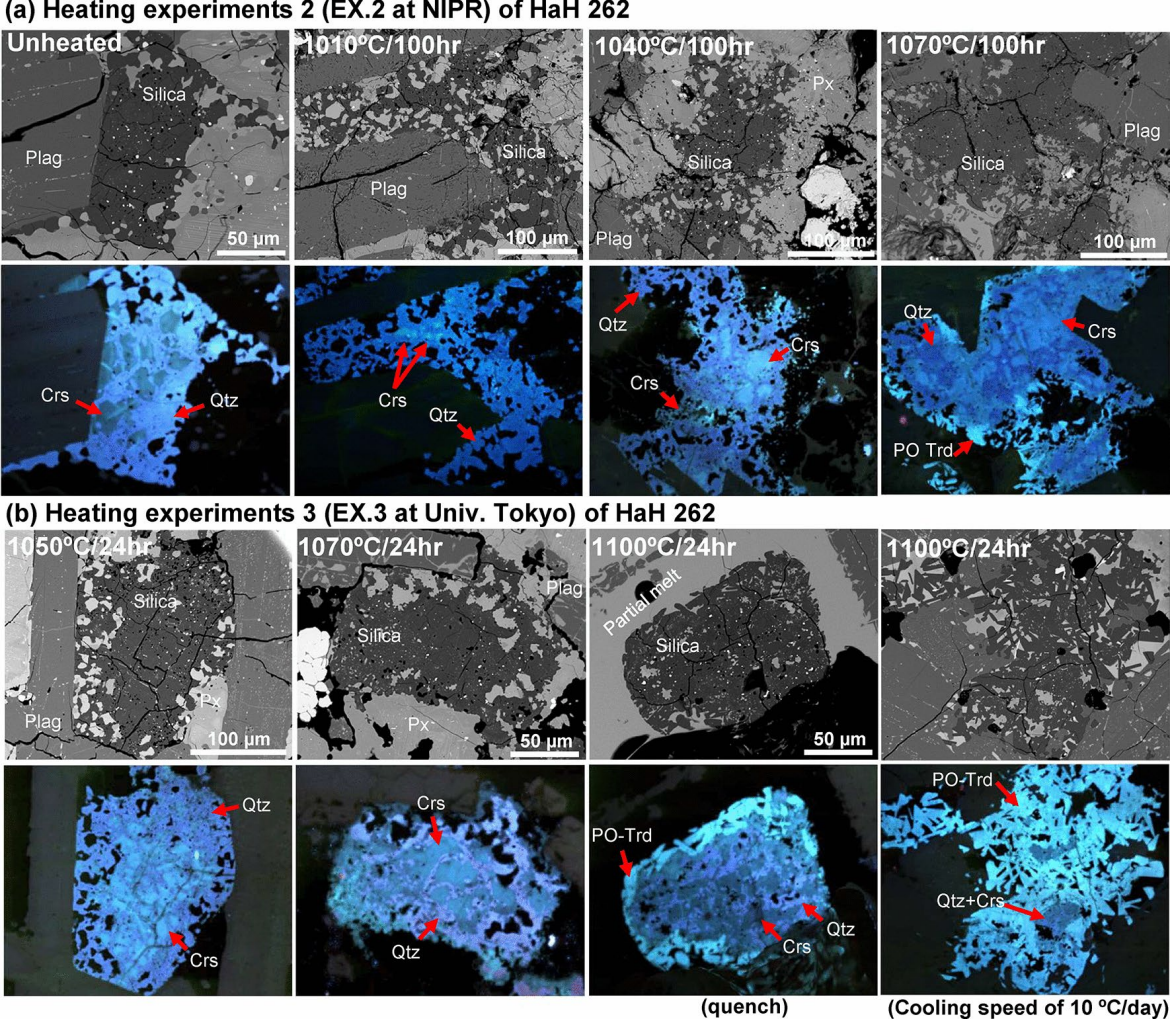
BSE and ChromaCL images of silica phases in unheated and heated HaH 262. (**a**) Run products of heating experiment 2 at NIPR, and (**b**) run products of heating experiment 3 at the Univ. Tokyo ^4^. The unheated and heated samples at 1010 °C for 100 h contain a large amount of quartz and minor cristobalite. However, the amount of cristobalite relative to quartz increases with a rise in temperature above 1040 °C. Furthermore, we found the crystallization of lathy PO-tridymite around silica grain in heated samples at 1070 °C for 100 h and 1100 °C for 24 h. Px = pyroxene; Plag = plagioclase; Qtz = quartz; Crs = cristobalite; PO-Trd = pseudo-orthorhombic tridymite.

To identify the silica phases in the samples, we used ChromaCL imaging and Raman spectral analysis (Fig. 1, Supplementary Fig. 8). The ChromaCL images display varied CL emission corresponding to the different silica phases. While CL color alone cannot be used directly to identify silica polymorphs, this imaging has a practical potential to distinguish the distribution of such polymorphs by recognizing changes in color tones. In this study, silica phases shown in all ChromaCL images were confirmed by Raman spectral analysis (e.g., Figs. 2, 3, 4, 5). CL color imaging combined with Raman spectroscopy allows for mapping two-dimensional distribution of silica phases with sufficient accuracy. We identified quartz, MC tridymite, cristobalite, MX tridymite, and silica glass in studied eucrites before heating. Additionally, we identified pseudo-orthorhombic (PO) tridymite in heated eucrites. Various tridymite modifications were identified by comparing the peak positions of Raman spectra with those previously reported^15^.

In Agoult, all silica phase shows a bright aqua-blue CL color, representing MC tridymite (Fig. 2), which shows short rods to rounded shapes (~ 100 µm in size). In A-87272, various CL colors are observed: bright aqua-blue for MC tridymite, dark blue for silica glass and MX tridymite, and purplish for quartz (Fig. 3). These phases coexist within the same lathy shaped silica grain (~ 500 µm in size), apparently consistent with a typical shape for MC tridymite in eucrites. Each silica grain in A-87272 comprises ~ 50 to ~ 100 vol% of silica glass and MX tridymite aggregates. Consequently, most of the MC tridymite in A-87272 convert into silica glass (probably diaplectic tridymite) and MX tridymite, with remnants of MC tridymite and quartz interspersed throughout the aggregate. But CL color imaging was not enough to distinguish individual phases well, resulting in no estimation of the amount ratios of the silica glass and MX tridymite. In HaH262, the silica minerals show purplish, and greenish CL colors, representing quartz and cristobalite, respectively (Fig. 5a). The coexisting quartz and cristobalite show irregular outlined aggregates with opaques minerals. Most cristobalites are rounded shape (~ 10 µm in size) and surrounded by quartz. The abundance of cristobalite for quartz in unheated HaH 262 is ~ 30 vol% at maximum.

### Isothermal heating experiments

All results in the heating experiments are summarized in Table 3. Each silica polymorph exhibits a different metamorphic response.

**Table 3:** Phase transition of silica polymorphs in our isothremal heating experiments.

Sample	Silica phase (Before heating)	Newly formed silica phase (After heating)							
		800 °C	900 °C	1000 °C	1010 °C	1040 °C	1050 °C	1070 °C	1100 °C
Agoult	MC tridymite	No phase trandsition			–	No phase trandsition	–	No phase trandsition	–
A-87272	Silica glass (diaplectic tridymite) + MX tridymite	–	Qtz	Qtz	–	Crs	-	Crs PO-trd	–
HaH 262	Quartz	–	–	–	No Phase transition	Crs	Crs	Crs PO-trd	Crs PO-trd

### Agoult

In Agoult, MC tridymite is only observed as a silica phase through all runs. When heated at 800–1070 °C for 100 h, the CL feature of all MC tridymite remained unchanged upon heating (Fig. 2), suggesting no recrystallization of MC tridymite into other silica polymorphs. This result contrasts with the previous reports^9^, where MC tridymite in Y 980433 recrystallized into quartz with similar heating condition.

### A-87272

Since unheated A-87272 contains several silica phases, the silica phases in same area were examined before and after heating (Fig. 3). The unheated A-87272 contains large aggregates of silica glass and MX tridymite, with small amounts of MC tridymite and quartz. For A-87272 heated at 800 °C for 100 h, the CL colors of silica phases appear unchanged, showing that the silica phases did not recrystallize into other polymorphs. However, in A-87272 heated at 900 and 1000 °C for 100 h each, the CL colors of the silica glass and MX tridymite aggregate partly changed from dark blue to purplish color, which we identified it as quartz. This newly formed quartz, with irregular shapes, appears interspersed within the aggregates. These quartz are frequently observed at the sample edges heated at 900 and 1000 °C (Supplementary Fig. 9), whereas such texture is absent in the unheated and heated at 800 °C samples. Such occurrences of the quartz are distinctly different from the original quartz, suggesting a recrystallization of silica glass and/or MX tridymite into quartz during heating. We also observed that silica glass coexists with MX tridymite in the sample heated at 900 °C, whereas the silica glass is not found in the sample heated at 1000 °C (Supplementary Fig. 10).

In A-87272 heated at 1040 and 1070 °C for 100 h each, we did not observe the same areas before and after heating. Regardless, we found characteristic occurrences of silica phases that were not present in the unheated sample. In A-87272 heated at 1040 °C for 100 h, the silica grains showed their cores with darker CL color and surrounding rims with slightly brighter CL color, identified as cristobalite and MX tridymite, respectively (Fig. 4). On the other hand, in A-87272 heated at 1070 °C for 100 h, the silica grains showed more complex structures, a cristobalite-core surrounded by MX tridymite and PO tridymite. The PO tridymite at the outermost rim shows the brightest aqua-blue CL color, while the MX tridymite shows the darkest CL color. The cristobalite at the core portions shows brighter CL color than the MX tridymite. The PO tridymite appears as aggregates of small lathy-shaped crystals, whereas the MX tridymite exists as a transitional layer between the PO tridymite and cristobalite. Additionally, the MX tridymite is characterized by the coexistence of inclusions probably derived from melts.

### HaH 262

We examined two sets of run products from Ex. 2 and 3 (Table. 2). For Ex. 2, HaH 262 was heated at 1010, 1040, and 1070 °C for 100 h each (Fig. 5a), whereas in Ex. 3, heating was done at 1050, 1070, and 1100 °C for 24 h each (Fig. 5b). The unheated HaH 262 contains abundant quartz and minor cristobalite. In HaH 262 heated at 1010 °C for 100 h shows no clear evidence for the phase transition of the silica phases. However, HaH 262 heated at ≥ 1040 °C contains a larger amount of cristobalite than unheated and heated at 1010 °C samples. The quartz in heated samples (e.g., 1070 °C for 24 h) shows an interstitial-like texture between the cristobalite particles. Moreover, in heated HaH 262 at 1070 °C for 100 h and 1100 °C for 24 h, we observed a lathy PO-tridymite around the rim of the silica grains. The PO-tridymite is more abundant in the sample that cools more slowly (at 10 °C/day), and forms its larger aggregates.

### Chemical compositions

**Fig. 6:**
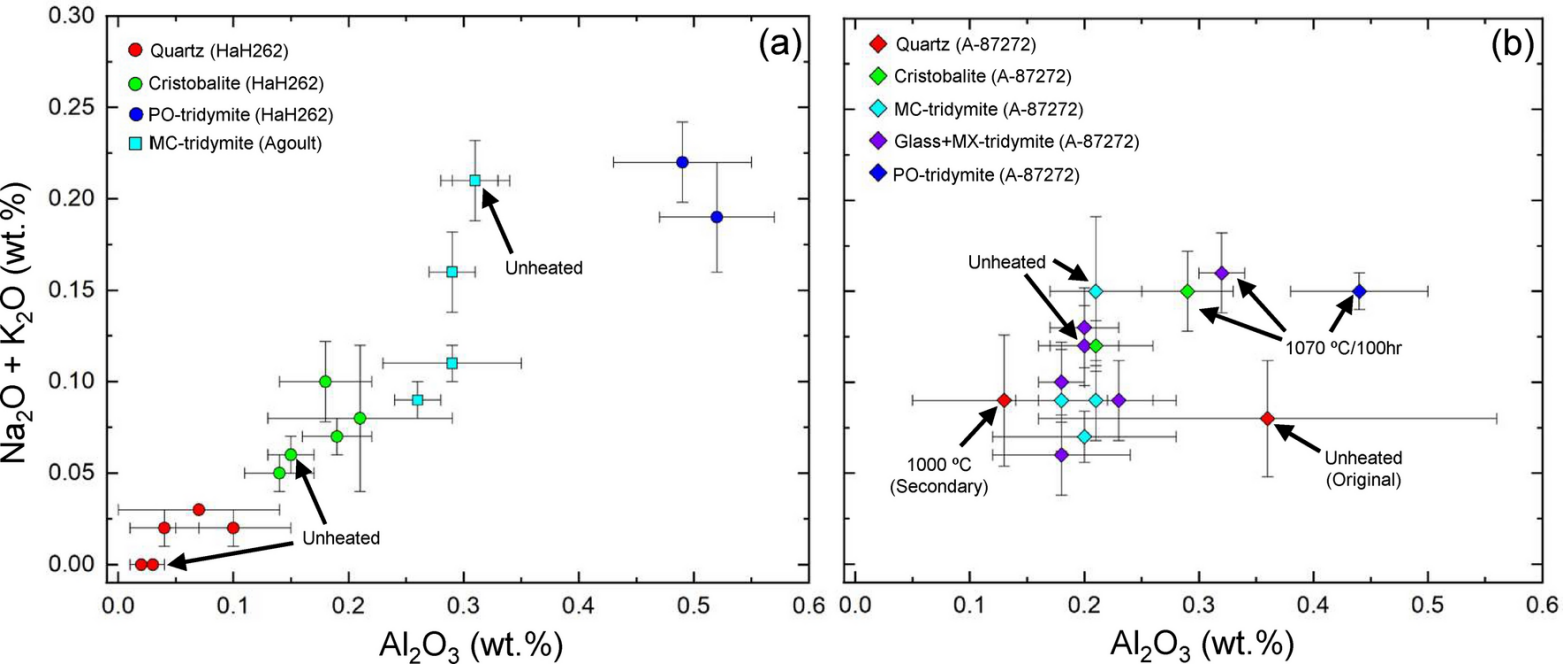
WDS analysis of Al_2_O_3_ for Na_2_O + K_2_O contains in silica phases. Error bars represent the 1σ error (wt%), calculated from quantitative values per analysis point. The detection limits for Al_2_O_3_, Na_2_O, and K_2_O by WDS analysis are less than 0.01 wt%. (**a**) Silica phases in Agoult and HaH 262 show positive correlation between Al_2_O_3_ and alkali elements (i.e., Na_2_O + K_2_O). In particular, the Al_2_O_3_ content of each silica phase characterizes the individual polymorphism. (**b**) The Al_2_O_3_ contents of MC tridymite and silica glass (+ MX tridymite) in A-87272 are plotted within the error bars. Secondary quartz within A-87272 heated at 1000 °C also has a composition similar to those of tridymite and silica glass, but different from those of original quartz.

The chemical compositions of the silica phases are summarized in Table 4 and Fig. 6. Each silica phase in HaH 262 and Agoult have distinct minor components like Al_2_O_3_ and alkali elements (Na_2_O, K_2_O) (Fig. 6a). Quartz is depleted in Al_2_O_3_ (< ~ 0.1 wt%) and alkali elements (Na_2_O + K_2_O =  < ~ 0.05 wt%), of which the amounts increase in the order of cristobalite (Al_2_O_3_ =  ~ 0.2 wt%; Na_2_O + K_2_O = ~ 0.07 wt%), MC tridymite (Al_2_O_3_ = ~ 0.3 wt%; Na_2_O + K_2_O =  ~ 0.15 wt%), and PO tridymite (Al_2_O_3_ = ~ 0.5 wt%; Na_2_O + K_2_O = ~ 0.20 wt%). On the other hand, MC tridymite, silica glass, and MX tridymite in unheated A-87272 have similar chemical compositions (Al_2_O_3_ = ~ 0.2 wt%; Na_2_O + K_2_O = ~ 0.13 wt%) within the error range (Fig. 6b). Quartz in the unheated A-87272 has abundant minor elements with a larger error than quartz in HaH 262. In the A-87272, since the distributions of MX tridymite and silica glass cannot be distinguished by CL color, they are combined as one component. Heated samples show similar Al_2_O_3_ levels (~ 0.2 wt%) across MC tridymite, silica glass (+ MX tridymite), and cristobalite, except at 1070 °C for 100 h. Secondary quartz in the heated A-87272 was too small size, and its composition was only obtained in the samples heated at 1000 °C. The quartz also indicates a composition similar to MC tridymite and silica glass, but the depletion in Al_2_O_3_ compared to the original quartz. Hence, the secondary quartz has different compositions of minor elements from original quartz, MC tridymite, silica glass and MX tridymite in both unheated and heated samples. Additional chemical data for pyroxene and plagioclase are provided in Supplementary Tables 1 and 2 and Supplementary Figs. 11 and 12.

**Table 4:** Chemical compositions of silica minerals in studied eucrites and quart-glass tube.

HaH262	Unheated		1010 °C		1040 °C		1050 °C		1070 °C			1100 °C			
	Qtz	Crs	Qtz	Crs	Qtz	Crs	Qtz	Crs	Qtz	Crs	PO-trd	Qtz	Crs	PO-trd	
Number	n = 4	n = 5	n = 5	n = 4	n = 7	n = 6	n = 5	n = 5	n = 4	n = 7	n = 6	n = 4	n = 4	n = 6	
SiO_2_	99.85 ± 0.21	100.91 ± 0.35	98.70 ± 0.19	98.47 ± 1.04	100.05 ± 0.23	100.69 ± 0.32	99.78 ± 0.25	101.08 ± 0.23	99.63 ± 0.86	100.23 ± 0.42	99.61 ± 0.78	99.76 ± 0.65	100.66 ± 0.54	100.02 ± 0.14	
TiO_2_	0.06 ± 0.01	0.07 ± 0.02	0.04 ± 0.04	0.07 ± 0.04	0.03 ± 0.02	0.07 ± 0.01	0.03 ± 0.01	0.06 ± 0.02	0.03 ± 0.02	0.06 ± 0.02	0.32 ± 0.08	0.06 ± 0.03	0.05 ± 0.02	0.17 ± 0.02	
Al_2_O_3_	0.02 ± 0.01	0.16 ± 0.02	0.04 ± 0.03	0.21 ± 0.08	0.03 ± 0.01	0.19 ± 0.03	0.03 ± 0.01	0.14 ± 0.03	0.10 ± 0.05	0.18 ± 0.04	0.52 ± 0.05	0.07 ± 0.07	0.15 ± 0.02	0.49 ± 0.06	
Cr_2_O_3_	< 0.02	0.03 ± 0.02	< 0.02	0.16 ± 0.04	< 0.02	0.16 ± 0.08	< 0.02	< 0.02	0.02 ± 0.01	0.05 ± 0.07	< 0.02	< 0.02	< 0.02	< 0.02	
FeO	0.12 ± 0.03	0.20 ± 0.06	0.41 ± 0.10	0.43 ± 0.05	0.34 ± 0.05	0.33 ± 0.05	0.51 ± 0.04	0.21 ± 0.01	0.33 ± 0.05	0.24 ± 0.12	0.46 ± 0.05	0.57 ± 0.10	0.38 ± 0.02	0.32 ± 0.03	
MnO	< 0.02	< 0.02	0.03 ± 0.01	< 0.02	< 0.02	< 0.02	< 0.02	< 0.02	< 0.02	< 0.02	< 0.02	< 0.02	< 0.02	< 0.02	
CaO	0.02 ± 0.01	0.03 ± 0.01	0.05 ± 0.02	0.02 ± 0.01	0.06 ± 0.02	0.07 ± 0.02	0.04 ± 0.01	0.02 ± 0.01	0.05 ± 0.03	0.04 ± 0.02	0.10 ± 0.01	0.05 ± 0.03	0.04 ± 0.01	0.08 ± 0.03	
MgO	< 0.01	< 0.01	< 0.01	< 0.01	< 0.01	< 0.01	< 0.01	< 0.01	< 0.01	< 0.01	< 0.01	< 0.01	< 0.01	< 0.01	
Na_2_O	< 0.01	0.04 ± 0.02	0.02 ± 0.01	0.06 ± 0.04	< 0.01	0.07 ± 0.01	< 0.01	0.05 ± 0.01	0.02 ± 0.01	0.08 ± 0.02	0.19 ± 0.03	0.03 ± 0.00	0.06 ± 0.01	0.14 ± 0.01	
K_2_O	< 0.01	< 0.01	< 0.01	0.02 ± 0.00	< 0.01	< 0.01	< 0.01	< 0.01	< 0.01	0.02 ± 0.01	< 0.01	< 0.01	< 0.01	0.08 ± 0.02	
Total	100.07	101.44	99.29	99.44	100.51	101.58	100.39	101.56	100.18	100.90	101.20	100.54	101.34	101.30	

A-87272	Unheated			800 °C		900 °C		1000 °C			1040 °C		1070 °C		
	Qtz	MC-Trd	Glass	MC-Trd	Glass + MX	MC-Trd	Glass + MX	Qtz	MC-Trd	MX-Trd	MX-trd	Crs	MX-trd	PO-trd	Crs
Number	n = 5	n = 6	n = 7	n = 5	n = 7	n = 6	n = 7	n = 9	n = 4	n = 5	n = 9	n = 4	n = 4	n = 4	n = 4
SiO_2_	100.10 ± 0.80	99.13 ± 0.79	100.32 ± 0.59	100.59 ± 0.36	100.02 ± 0.31	98.68 ± 0.53	98.91 ± 0.74	98.50 ± 0.31	99.90 ± 0.40	98.63 ± 0.51	100.43 ± 0.19	98.68 ± 0.31	99.19 ± 0.13	99.68 ± 0.26	99.26 ± 0.46
TiO_2_	0.05 ± 0.03	0.09 ± 0.03	0.09 ± 0.02	0.06 ± 0.03	0.06 ± 0.02	0.06 ± 0.03	0.05 ± 0.03	0.04 ± 0.03	0.09 ± 0.03	0.05 ± 0.02	0.09 ± 0.02	0.07 ± 0.05	0.09 ± 0.03	0.09 ± 0.03	0.07 ± 0.03
Al_2_O_3_	0.36 ± 0.20	0.21 ± 0.04	0.20 ± 0.03	0.20 ± 0.08	0.23 ± 0.05	0.18 ± 0.04	0.18 ± 0.02	0.13 ± 0.08	0.21 ± 0.05	0.20 ± 0.03	0.18 ± 0.06	0.21 ± 0.05	0.32 ± 0.02	0.44 ± 0.06	0.29 ± 0.04
Cr_2_O_3_	< 0.02	< 0.02	< 0.02	< 0.02	< 0.02	< 0.02	< 0.02	< 0.02	< 0.02	0.03 ± 0.01	< 0.02	0.04 ± 0.02	0.02 ± 0.01	< 0.02	< 0.02
FeO	0.30 ± 0.19	0.10 ± 0.04	0.26 ± 0.05	0.09 ± 0.06	0.14 ± 0.07	0.34 ± 0.13	0.27 ± 0.05	0.43 ± 0.43	0.27 ± 0.05	0.23 ± 0.03	0.27 ± 0.05	0.29 ± 0.04	0.36 ± 0.02	0.54 ± 0.07	0.31 ± 0.02
MnO	< 0.02	< 0.02	< 0.02	< 0.02	< 0.02	< 0.02	< 0.02	0.04 ± 0.03	< 0.02	< 0.02	< 0.02	< 0.02	0.04 ± 0.02	< 0.02	< 0.02
CaO	0.04 ± 0.01	0.05 ± 0.02	0.06 ± 0.01	0.04 ± 0.02	0.03 ± 0.01	0.09 ± 0.03	0.08 ± 0.01	0.07 ± 0.03	0.05 ± 0.01	0.06 ± 0.02	0.09 ± 0.01	0.03 ± 0.01	0.04 ± 0.00	0.11 ± 0.02	0.04 ± 0.01
MgO	< 0.01	< 0.01	< 0.01	< 0.01	< 0.01	< 0.01	< 0.01	< 0.01	< 0.01	< 0.01	< 0.01	< 0.01	< 0.01	< 0.01	< 0.01
Na_2_O	0.02 ± 0.01	0.02 ± 0.01	0.02 ± 0.01	0.03 ± 0.01	0.03 ± 0.01	0.06 ± 0.02	0.06 ± 0.02	0.05 ± 0.02	0.06 ± 0.02	0.07 ± 0.02	0.03 ± 0.01	0.09 ± 0.01	0.12 ± 0.01	0.12 ± 0.00	0.12 ± 0.02
K_2_O	0.06 ± 0.03	0.13 ± 0.04	0.10 ± 0.02	0.04 ± 0.01	0.06 ± 0.02	0.03 ± 0.02	0.04 ± 0.01	0.04 ± 0.03	0.03 ± 0.01	0.06 ± 0.01	0.03 ± 0.02	0.03 ± 0.02	0.04 ± 0.02	0.03 ± 0.01	0.03 ± 0.01
Total	100.93	99.73	101.05	101.05	100.57	99.50	99.59	99.30	100.63	99.00	101.12	100.44	100.18	101.01	100.12

Agoult	Unheated	800 °C	900 °C	100 °C	1040 °C	1070 °C		Quartz-tube	Unheated	1010 °C	1040 °C		1070 °C		
	MC-Trd	MC-Trd	MC-Trd	MC-Trd	MC-Trd	MC-Trd			Glass	Glass	Glass	Crs	Glass	Crs	
Number	n = 5	n = 5	n = 7	n = 6	n = 4	n = 7		Number	n = 4	n = 4	n = 4	n = 4	n = 4	n = 4	
SiO_2_	99.07 ± 0.20	99.67 ± 0.31	100.04 ± 0.76	99.52 ± 0.56	100.08 ± 0.17	99.03 ± 0.29		SiO2	98.96 ± 0.05	98.85 ± 0.19	99.26 ± 1.10	99.12 ± 0.32	99.38 ± 0.12	99.38 ± 1.11	
TiO_2_	0.10 ± 0.05	0.11 ± 0.01	0.08 ± 0.02	0.12 ± 0.01	0.08 ± 0.01	0.10 ± 0.02		TiO2	< 0.03	< 0.03	< 0.03	< 0.03	< 0.03	< 0.03	
Al_2_O_3_	0.31 ± 0.02	0.31 ± 0.03	0.26 ± 0.02	0.29 ± 0.02	0.29 ± 0.06	0.29 ± 0.02		Al2O3	< 0.01	< 0.01	< 0.01	< 0.01	< 0.01	0.02 ± 0.01	
Cr_2_O_3_	< 0.02	< 0.02	< 0.02	< 0.02	< 0.02	< 0.02		Cr2O3	< 0.02	< 0.02	< 0.02	< 0.02	< 0.02	< 0.02	
FeO	0.37 ± 0.12	0.25 ± 0.04	0.36 ± 0.13	0.24 ± 0.09	0.40 ± 0.16	0.28 ± 0.12		FeO	< 0.02	0.03 ± 0.01	< 0.02	0.05 ± 0.01	< 0.02	< 0.02	
MnO	< 0.02	< 0.02	< 0.02	< 0.02	< 0.02	< 0.02		MnO	< 0.02	0.04 ± 0.01	0.03 ± 0.01	0.03 ± 0.01	0.03 ± 0.01	< 0.02	
CaO	0.04 ± 0.01	0.04 ± 0.01	0.06 ± 0.02	0.05 ± 0.01	0.07 ± 0.04	0.05 ± 0.01		CaO	< 0.01	< 0.01	< 0.01	< 0.01	< 0.01	< 0.01	
MgO	< 0.01	< 0.01	< 0.01	< 0.01	< 0.01	< 0.01		MgO	< 0.01	< 0.01	< 0.01	< 0.01	< 0.01	< 0.01	
Na_2_O	0.05 ± 0.01	0.07 ± 0.01	0.09 ± 0.01	0.11 ± 0.02	0.11 ± 0.01	0.14 ± 0.02		Na2O	< 0.01	< 0.01	< 0.01	< 0.01	0.02 ± 0.02	0.02 ± 0.00	
K_2_O	0.16 ± 0.02	0.14 ± 0.02	< 0.01	0.05 ± 0.01	< 0.01	0.02 ± 0.01		K2O	< 0.01	< 0.01	< 0.01	< 0.01	0.02 ± 0.01	< 0.01	
Total	100.10	100.59	100.89	100.38	101.03	99.91		Total*	98.96	98.92	99.29	99.20	99.45	99.42

## Discussion

According to the phase diagram for silica minerals^5,6^, the thermodynamically stable field for quartz, tridymite, and cristobalite at 1 atm are < ~ 870 °C, ~ 870 to ~ 1470 °C, and 1470 ~ 1727 °C, respectively. In eucrites, silica minerals crystallize at the solidus temperature (~ 1060 °C) during the last stage of eucritic magma crystallization^14^. Although this temperature falls within the stable field for tridymite, various silica polymorphs are found in eucrites^7,9–11^. It shows that the presence of various silica polymorphs in eucrites is likely influenced by secondary processes such as thermal and shock metamorphism after the silica phases crystallize on the parent body. Our experiments revealed that the phase transition of silica polymorphs is influenced not only by temperature but also by the initial silica phase as discussed below.

**Fig. 7:**
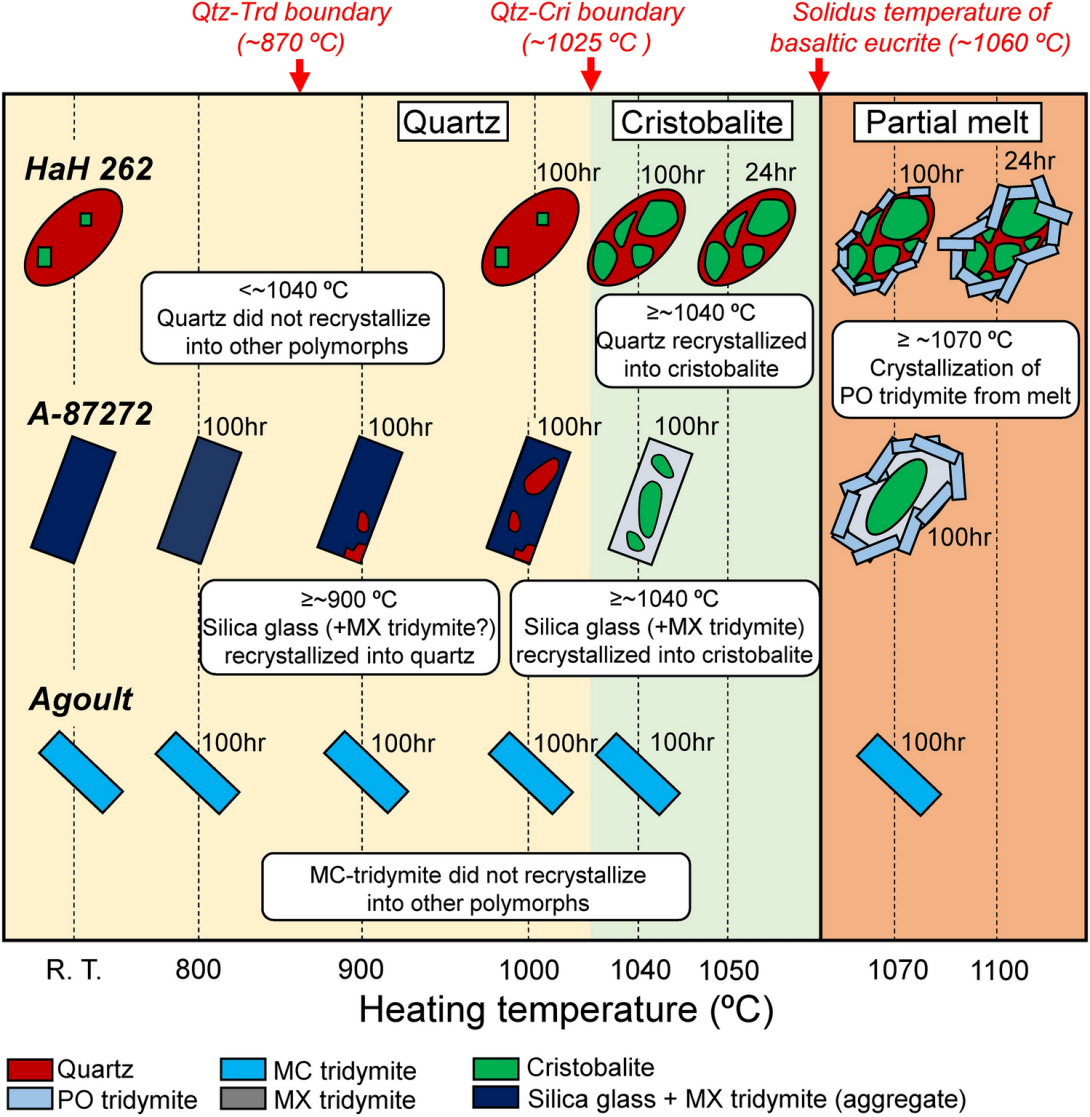
Schematic diagram on combined results from the isothermal experiments of basaltic eucrites studied here. Silica polymorphs show a different behavior at heating temperatures. MC tridymite in Agoult did not recrystallize into other polymorphs on heating to 1070 °C. Quartz in HaH 262 partially recrystallized into cristobalite on heating above 1040 °C. The aggregate composed by silica glass and MX tridymite partially recrystallized into quartz with heating above 900 °C. Moreover, the aggregates partially recrystallized into cristobalite with heating above 1040 °C. PO-tridymite was characteristically observed around silica grains in HaH 262 and A-87272 heated at above 1070 °C, where it was crystallized from partial melt.

Our experimental results are summarized in Fig. 7. The yellow and green fields represent the stable fields for quartz and cristobalite, respectively. The right portion in this figure is divided by the eucrite solidus temperature at ~ 1060 °C^14^, in red. The stable field for tridymite is not shown, as tridymite is not a pure silica mineral. The tridymite in Agoult may metastably exist within the stable fields of quartz and cristobalite. This suggestion is based on this study that the aggregate composed of silica glass in A-87272, containing minor elements similar to the tridymite in Agoult, partially recrystallized into quartz or cristobalite during heating. Although the quartz-cristobalite boundary has been previously estimated at ~ 1025 °C^16,17^, we have also adopted the quartz-cristobalite boundary at 1025 °C based on our experiments here. Whereas in the HaH 262 and A-87272 heated at ≥ 1070 °C, PO tridymite is recognized. These PO tridymite are considered to have crystallized from the melt. Hence, it is important to note that these PO tridymite have undergone a different formation process than other phases produced by solid–solid phase transition (recrystallization). The following sections provide a detailed interpretation of the phase transition processes of each silica phases.

One of the significant findings in this study is the solid–solid phase transition from quartz into cristobalite within the stability field of tridymite. In HaH 262 heated at ≤ 1050 °C, no tridymite was observed, indicating that recrystallization of quartz and cristobalite in eucrites do not recrystallize into tridymite via solid–solid phase transition. A reasonable explanation for the absence of tridymite is the low abundance of impurities in quartz and cristobalite because impurities are essential for recrystallization into tridymite. It is well known that tridymite is not a pure silica mineral, and its synthesis usually requires an abundant flux and/or mineralizer, such as alkali components^18,19^. Thus, previous studies have successfully synthesized tridymite from quartz by adding a few wt% of alkali components^16–21^, but the impurities in quartz and cristobalite in HaH 262 (~ 0.5 wt%) might be insufficient for recrystallization into tridymite. Another possibility responsible for the absence of tridymite could be the slow recrystallization rate of quartz and cristobalite into tridymite; according to the Ono model, this requires a slow cooling rate of less than 0.1 °C/hr^9^.

On the other hand, in HaH 262, recrystallization from quartz to cristobalite was not observed at 1010 °C but was evident at temperatures ≥ 1040 °C. A similar result was obtained in the observation of quartz-glass tubes used in Ex. 2 (Supplementary Fig. 13). The recrystallization from quartz and/or silica glass into cristobalite within the stable field of tridymite has been well documented in heating experiments with pure silica^16,19,20^. Therefore, the quartz in HaH 262 may similarly behave to pure silica during heating, suggesting that the quartz-cristobalite boundary for eucrites occurs between 1010 and 1040 °C. This temperature range corresponds well with the previously suggested quartz-cristobalite boundary at ~ 1025 °C^16^. In contrast to the fact that quartz recrystallizes into cristobalite at ≥ 1040 °C, we found no clear evidence of recrystallization from cristobalites into quartz in the samples heated at 1010 °C. Because there is abundant quartz around the cristobalite in HaH 262 before heating, making it unsuitable for observing the cristobalite to quartz recrystallization.

Another significant finding in this study is the difficulty of the phase transition from tridymite into other silica phase below the sub-solidus temperature of eucrite. This suggestion is based on the fact that MC tridymite in Agoult did not recrystallize into other silica polymorphs during any heating experiments. In previous heating experiment suggests that MC tridymite recrystallized into quartz (i.e., Ono model). This suggestion is based on that MC-tridymite in Y 980433 recrystallized into quartz during an isothermal experiment at 800 °C for 96 h^9^. This result is different from our experimental result for Agoult heated at 800 °C for 100 h. Here, we point out the two potential issues with the previous heating experiments. (1) In Y 980433 is a shock degree D eucrite^3^, where we observed a large amount of silica glass and MX tridymite coexistence with MC tridymite (Supplementary Fig. 14). (2) Y 980433 originally contains quartz in the sample before the heating process^11^, making it difficult to distinguish this original quartz from newly-formed quartz. Consequently, it is unclear whether the recrystallization from MC tridymite into quartz really occurred or not. Therefore, Agoult is believed to be an ideal starting material for verifying the problem because all silica phases present within it are MC tridymite. The MC tridymite shows finer-grained crystals compared with that in Y 980433. More rapid progress of the recrystallization is expected for the MC tridymite in Agoult than in the MC tridymite of Y 980433, if the recrystallization from tridymite into quartz could actually occur. Additionally, Agoult is characterized by a shock degree A, suggesting that it has no structural deformation within the crystal. Based on our experiments, the recrystallization of MC tridymite into quartz by thermal metamorphism is not thought to be a plausible process in eucrites. On the other hand, it is unclear whether up to 100 h heating is sufficient for the phase transition from tridymite into quartz. However, the fact that absence of quartz in many cumulate eucrites^11^, which have extremely slow cooling rates, indicates that tridymite cannot recrystallize into quartz. Moreover, ChromaCL imaging and Raman spectra analyses allow for high-spatial observation of silica phases, enabling the detection of minor phase transitions in a few µm scale. Hence, it is inferred that quartz in Y 980433 reported in previous heating experiments may be possibly quartz that existed before heating.

**Fig. 8:**
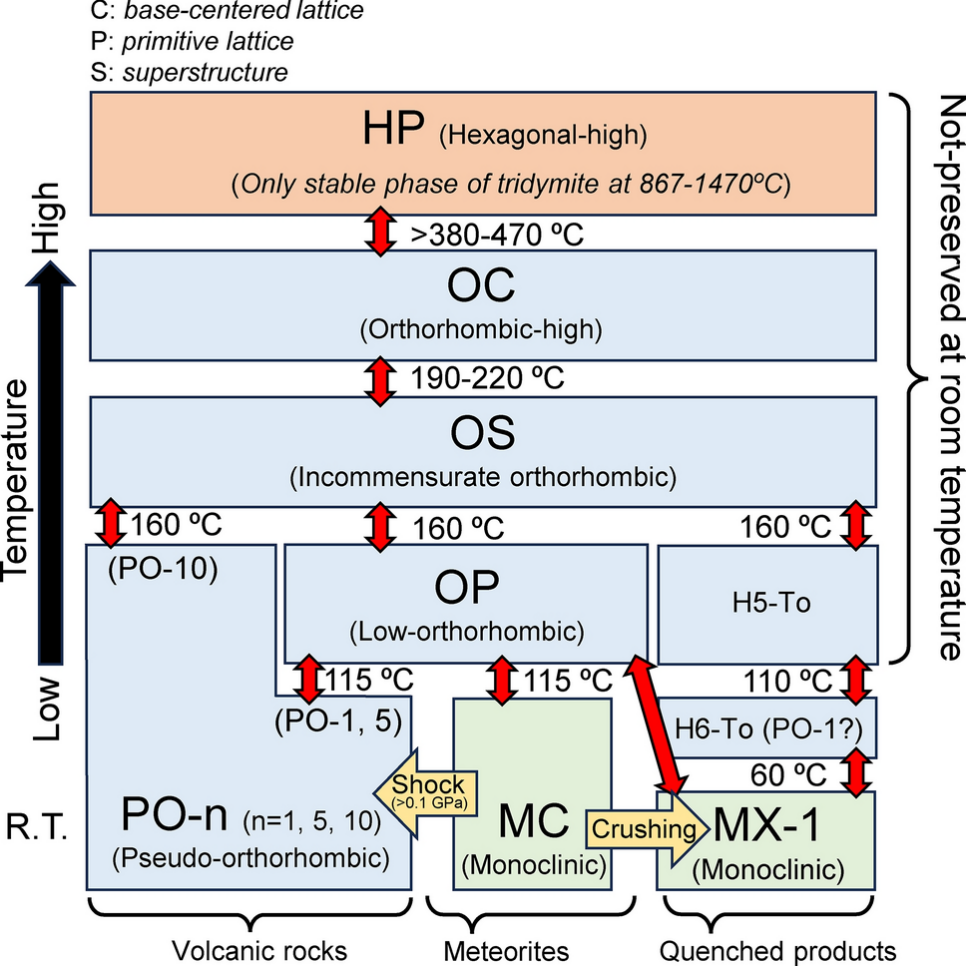
Modifications of tridymite at different temperatures. This figure is modified after previous research ^9^, based on several related works ^22–26^. MC, PO-n, and MX are metastable phases observed in room temperature. Each transition temperature is known to vary depending on the amount of impurities, showing supposed values here. Moreover, the phase transitions of tridymite during heating might be affected by the origin of the starting materials.

Tridymite has many modifications at temperatures below ~ 470 °C (Fig. 8)^22–25^. Three metastable modifications are commonly reported so far at room temperature: MC, PO-n (where n = 1, 2, 5, 10), and MX-1. MC tridymite is dominant in ≥ type 5 basaltic eucrites and cumulate eucrites^9,11^, while PO-n is prevalent in volcanic rocks on Earth. MX-1 has not been reported in meteorites, but we now find out it for the first time in shocked eucrites (i.e., A-87272 and Y 980433). In this study, we found MC tridymite in Agoult. No other its modifications were found. Similarly, the previous heating experiments reported that MC tridymite in Y980433 heated at 500 °C for 168 h and 800 °C 96 h did not recrystallize into other modifications, suggesting that this non-recrystallization process of MC tridymite may be derived from the difference in starting materials^9^. So far, such characteristic behavior of MC tridymite when heating has not been known. However, it should be convincing that MC tridymite in eucrites tends not to easily recrystallize into other modification.

The silica glass in A-87272 is diaplectic tridymite because its chemical composition is similar to the surrounding MC tridymite (Fig. 6b). Furthermore, it has a lathy shape, which represents a typical euhedral shape for MC tridymite found in eucrites^10^. Moreover, this silica glass coexists with MX tridymite. MX tridymite is formed when MC tridymite is stressed such as crushing in a mortar^26^. Thus, the presence of MX tridymite with silica glass implies that the MX tridymite corresponds to the transition phase that occurred during the conversion from MC tridymite into silica glass by shock metamorphism.

Another significant finding in this study is that the shock-induced silica glass and MX tridymite aggregates in A-87272 partially recrystallized into quartz when heated at 900 and 1000 °C and partially into cristobalite at ≥ 1040 °C (Figs. 3, 4). It is not clear whether silica glass or MX tridymite is involved in the recrystallization during heating. However, the absence of silica glass in the samples heated at ≥ 1000 °C suggests that silica glass preferentially has a role in the recrystallization. However, this assumption does not rule out the recrystallization of MX tridymite into quartz or cristobalite. The observed newly formed quartz is significantly different in occurrence from the original quartz in unheated A-87272. Additionally, in both experiments at 900 and 1000 °C, no quartz originally exist in the area where quartz was newly formed by heating (Fig. 3). This clearly shows a recrystallization of quartz from the aggregate composed of silica glass and MX tridymite. On the other hand, the cristobalite in the heated samples at ≥ 1040 °C is also considered to be recrystallized from the aggregates because cristobalite was originally absent in A-87272. The newly formed quartz and cristobalite have an impurity abundance similar to silica glass, MX tridymite and MC tridymite in unheated sample. Furthermore, these silica glass, MX tridymite and MC tridymite in unheated sample have the abundance of impurities within the MC tridymite in the typical eucrites (Fig. 6). The facts imply that the abundances of impurities in the silica glass is not sufficient to promote the recrystallization from silica glass into tridymite via solid-state phase transition process. The slightly lower abundance of Al_2_O_3_ in newly formed quartz in A-87272 heated at 1000 °C compared to silica glass and cristobalite may reflect the denser crystal structure of the quartz, which should excrete the Al_2_O_3_ component during recrystallization. This indicates that once silica glass and MX tridymite formed from MC tridymite by shock metamorphism, a portion of them can recrystallize into quartz and/or cristobalite through subsequent post-shock annealing. The recrystallization of aggregates composed of silica glass and MX tridymite into quartz at 900–1000 °C and into cristobalite at ≥ 1040 °C is consistent with the observation in HaH 262 where the quartz recrystallizes into cristobalite at ≥ 1040 °C.

The evidence supporting the above discussion has been recognized in natural eucrites. Juvinas (basaltic eucrite) has two distinct lithological features: coarse-grained and fine-grained portions. Within the coarse-grained portion, which is considered lithologically to experience shock partial-melting^27,28^, a complex intergrowth of MC tridymite and quartz is present (Supplementary Fig. 15), suggesting that the quartz and MC tridymite can coexist during a heating process where a part of the pre-existing MC tridymite converts into silica glass and MX tridymite by shock event. These silica glass and MX tridymite recrystallized into quartz as a result of intense post-shock annealing, which also induced a partial melting. Such characteristics can be only observed in the coarse-grained portion of Juvinas that experienced a shock partial-melting, and has not been found in other eucrites.

The last significant finding in this study is the crystallization of PO tridymite from the quenched melt above the solidus temperature of eucrite. In the samples heated at ≥ 1070 °C except for Agoult, PO tridymite was mainly observed around the rims of silica particles (Figs. 4, 5). These PO tridymite are euhedral lath-shaped and interstitially surrounded by melt. This observation suggests that the rims of the silica particles once partially melted, and subsequently, PO tridymite directly crystallized from that melt. This is further supported by the fact that this PO tridymite contains more abundant impurities, such as alkali elements than other silica phases (Fig. 6). Here, comparing samples of A-87272 heated at 1040 °C (unmelted) and 1070 °C (partially melted), small inclusions are present in the MX tridymite portion only in the sample heated at 1070 °C. This texture may be associated with the reaction front between the original MX tridymite rim and the partial melt. Moreover, the presence of interstitial melt surrounding the PO tridymite suggests that the PO tridymite crystallized earlier than the melt. Its particular feature can be explained by the presence of immiscible silica-rich melt around silica particles. Such immiscible melts can also experimentally form from slow-cooled eucritic melts^29^. According to their experiment, the residual melts of eucrite are divided into liquid 1 (enriched in Fe, Ti, Ca, P, Ba, S, F and Cl) and liquid 2 (enriched in Si, K and Al) at the last stage of the crystallization process. The presence of the liquid 2 involved in immiscible Si and alkali-rich melt, suggests that it assists in the crystallization of tridymite in an equilibrium state. Likewise, such melts are considered to be solidified at higher temperatures than the surrounding melts.

The absence of cristobalite at the rims of silica particles and an increase in the amount of lath-shaped PO tridymite with slow cooling suggest that tridymite directly crystallized from the silica-rich melt until the solidification of the melt. The Ono model proposed that the primary silica phase crystallized from eucritic magma is cristobalite, based on the observation showing an intergrowth texture with surrounding plagioclase and pyroxene^9^. The occurrence suggests that these cristobalites simultaneously crystallized from eucritic melt with pyroxene and plagioclase at solidus temperatures of eucrite. However, our experiments result in the possibility that tridymite can crystallize as the primary phase from eucritic magma. From these observations, it is suggested that in eucritic melts where an immiscible melt is not formed, cristobalite crystallizes as an unequilibrium phase at the solidus temperature, whereas, when an immiscible silica-rich melt is formed, tridymite crystallizes as an equilibrium phase from the melt until the surrounding melt begins to solidify.

In conclusion, this study experimentally demonstrates the complex phase transition processes of silica phases and suggests a new pathway for silica mineral formation in eucrites. Three significant results were obtained below the sub-solidus temperature of eucrites: (1) Quartz recrystallizes into cristobalite at ≥ 1040 °C; (2) MC tridymite does not recrystallize into other polymorphs; (3) Shock-induced silica glass recrystallizes into quartz at 900–1010 °C and into cristobalite at ≥ 1040 °C. These findings indicate that MC tridymite in eucrites remains stable during reheating and does not form from other silica phases below the solidus temperature. The strong stability of tridymite, particularly for secondary heating, is consistent with the abundant presence of tridymite in cumulate eucrites. However, tridymite can transform into quartz or cristobalite via silica glass through shock metamorphism and post-shock heating. Additionally, we found that PO tridymite directory crystallizes from quenched melts. Previously, while cristobalite has been considered as the initial silica phase, which crystallized from eucritic magma, we show that the first crystallizing silica minerals may not always be cristobalite. This fact suggests the possibility of the formation of tridymite in more eucrites. In fact, tridymite in several unequilibrated basaltic eucrites (i.e., type 1 and 2) has been reported^30^. The Ono model cannot explain the presence of tridymite in such eucrites. Hence, our findings not only necessitate a revision of the simple silica mineral models previously proposed for eucrites but also suggest the need for a new silica mineral formation model. In order to construct such a new model, we need a systematic observation of silica minerals in natural eucrites, which remains a topic for future investigation. Since silica minerals are relatively rare in planetary materials, but have been recognized as component minerals in other achondrites such as lunar and Martian meteorites^12,31^, our results may also be applicable to the comparative studies on the formation of silica minerals among different bodies.

## Methods

We used an optical microscope, a field emission scanning electron microscope (FE-SEM: JEOL JSM-7100) equipped with an energy dispersive spectrometer (EDS: Oxford AZtec Energy) and a color-cathodoluminescence imaging system (ChromaCL2: GATAN) at NIPR. We used Raman spectroscopy using an inVia Raman microscope (Renishaw) at NIPR and NRS-5100 (JASCO) at ISAS/JAXA. Mineral chemistry was investigated using a Field-emission electron probe microanalyzer (FE-EPMA: JEOL JXA-iHP200f.) at ISAS/JAXA. Acceleration voltage of 15 kV, beam current of 10 nA, and beam spot diameter of 1 µm were used for all measurements of the minerals studied here (i.e., silica minerals, pyroxene, and plagioclase). Natural and synthetic phases (Micro-Analysis Consultants Ltd.) were used as standards, and the data obtained were corrected using ZAF program.

In this study, three types of run products were used: two of them were prepared through our experiments (Experiments 1 and 2), while one was prepared in a prior study (Experiment 3^4^). Details of the experimental conditions and run products are summarized in Table 2 and below.

### Experiment 1 (EX. 1 at ISAS)

**Fig. 9:**
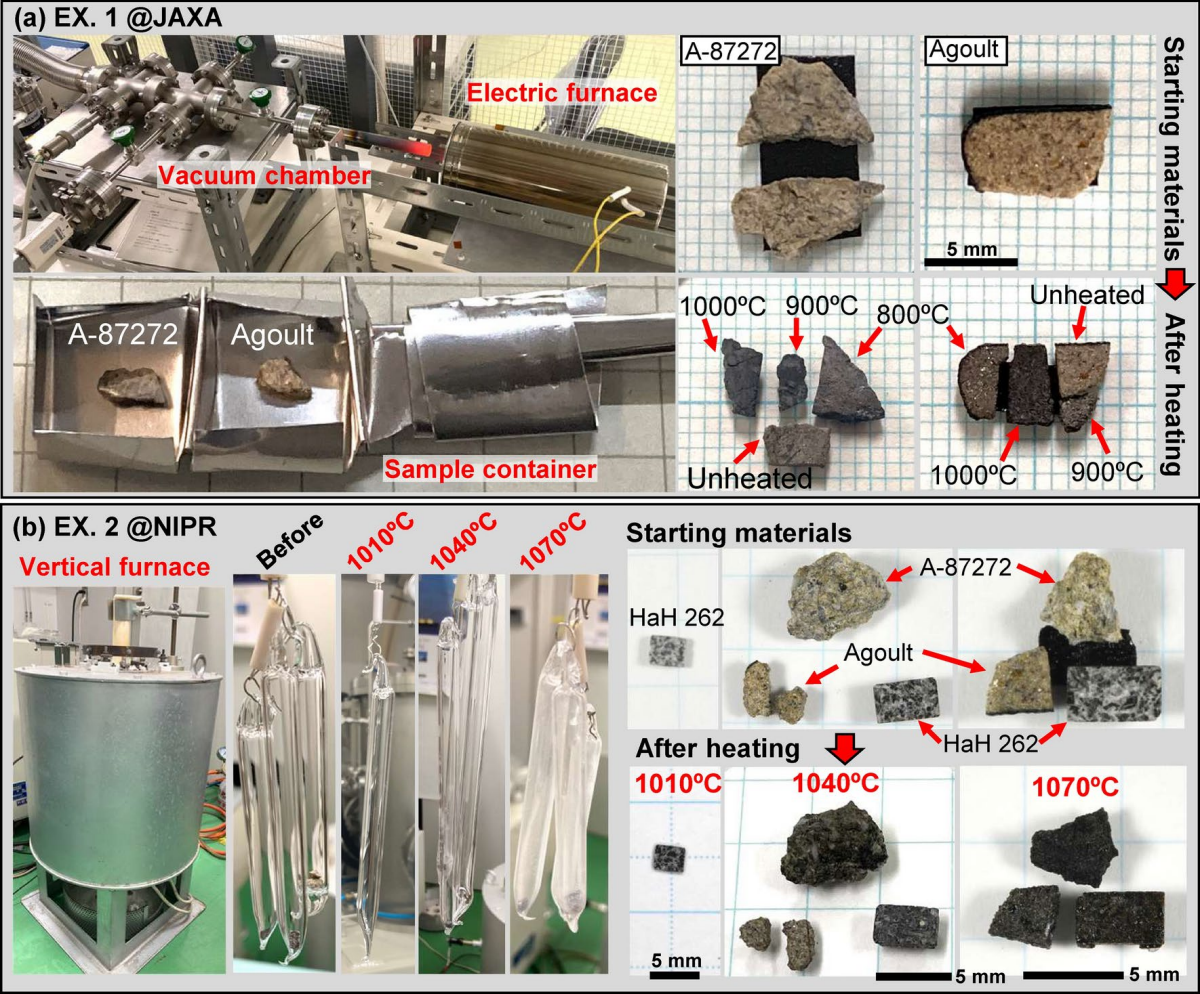
Experimental set-up for isothermal heating of basaltic eucrite chips. (**a**) The heating system consists of a chamber built with turbo-molecular vacuum pump, rotary pump, vacuum gauge, and electric furnace (EX. 1 @ISAS, JAXA). A Pt–Rh thermocouple was held right above the chamber. The sample container was evacuated to ~ 10^−6^–10^–5^ Pa. Small chips of eucrites were placed in a stainless board sample container. This system was used for the experiments at 800, 900, and 1000 °C for 100 h. (**b**) The vertical furnace was used for the isothermal experiments at 1010, 1040 and 1070 °C for 100 h (EX. 2 @NIPR). The samples were vacuum-sealed in a quartz-glass tube using a rotary vacuum pump and heated by a vertical furnace.

Three heating experiments ranging from 800 to 1000 °C for A-87272,79 and Agoult were conducted using the electric furnace at ISAS/JAXA (Fig. 9a)^32^. The sample chips were placed inside a sample container made of stainless board, which was evacuated down to approximately 1.6 × 10^–6^–1.9 × 10^–5^ Pa using a turbo-molecular pump. Heating temperatures within the furnace were monitored by a Pt–Rh thermocouple, held right above the chamber. Three types of heating experiments were carried out: at 800 °C for 100 h, 900 °C for 100 h, and 1000 °C for 100 h. After heating, the samples were left at room temperature in the vacuum chamber.

For A-87272, the following procedure was used to observe the same area before and after the heating process. (1) Slices of A-87272 (~ 10 mm × ~ 2 mm) were separated from the larger chips using by a diamond saw. (2) The surfaces of the slices were polished using lapping films (3 M). (3) The polished slices were then coated with osmium (Os) for petrographic observation. (4) After the petrographic observation, the Os coating was removed with lapping films. (5) Silica grains observed via the SEM and CL imaging were further characterized through Raman spectral analysis. (6) The polished slice was subsequently divided into smaller fragments. (7) These fragments were then subjected to three types of heating experiments. (8) Heated samples (run products) were observed to examine petrographic features for clarifying potential effects from the heating.

### Experiment 2 (EX. 2 at NIPR)

Three heating experiments ranging from 1010 to 1070 °C were conducted using a tubular-vertical furnace at NIPR^33^ (Fig. 9b). Heating temperatures were controlled by an R-type thermocouple (Pt100-Pt87Rd13 pair) inserted from the side of samples. A-87272,88, Agoult, and HaH 262 were heated at 1040 °C and 1070 °C for 100 h each. In addition, HaH 262 was heated at 1010 °C for 100 h. The sample chips were vacuum-sealed (~ 10^–1^ Pa) in a quartz-glass tube using a rotary vacuum pump. After heating, the samples were left in the quartz tube at room temperature. We also observed the quartz-glass tubes used in the experiments because it is comparable as a heating experiment for pure silica. The quartz-glass tubes were partially devitrified by heating above 1040 °C, where cristobalite formed on the outer and inner wall of a quartz-glass tube (Supplementary Fig. 13).

### Experiment 3 (EX. 3 at Univ. Tokyo)

Heating experiments of HaH 262 were performed by Yamaguchi and his colleagues^4^, showing that the HaH 262 were heated for a duration of 24 h at 1050, 1070, and 1100 °C and then quenched in air. These run products were placed inside the Pt foil suspended in an alumina tube of a vertical furnace with 1-bar mixed gas at the University of Tokyo. A gas mixture of CO_2_–H_2_ was used to control the oxygen fugacity at 1 log unit below the iron– wüstite buffer (log fO_2_ = IW − 1) for all runs. In this study, these samples were employed here.

### Sample descriptions

We selected three basaltic eucrites for our study: Asuka 87272 (A-87272) (,79 and ,88), Agoult, and, Hammada al Hamra 262 (HaH 262) as shown in Table 1. A-87272 was provided from the National Institute of Polar Research, Tokyo (NIPR), while Agoult was acquired from meteorite dealers. We obtained the chips and run products of HaH 262, which were used in previous heating experiments^4^ to observe petrographic features in this study. For our heating experiments, we prepared rock slices and polished thick sections (PTS). The petrologic types^2,13^ and shock degrees^3^ were determined from the PTS, as shown in Table 1.

### Agoult (type 5, shock degree A)

Agoult is composed of a fine-grained mineral assemblage (Supplementary Fig. 3) with mineral boundaries displaying 120° triple junctions. Detailed mineralogical and geochemical studies on Agoult have been conducted by several previous investigations^34,35^. The low-Ca pyroxene in Agoult has thin augite lamellae (< ~ 1 µm thick) that is homogeneously distributed within host grains (i.e., type 5^2^). Both pyroxene and plagioclase show a sharp (< 2°) optical extinction under the cross-polarized light (i.e., shock degree A^3^).

### A-87272 (type 7, shock degree E)

A-87272 is an unbrecciated basaltic eucrite, and shows coarse-grained subophitic textures and minor fine-grained portions (Supplementary Fig. 2). Thin augite lamellae are located along the rim but not in the core (i.e., remnant Ca-zoning). These mineralogical features of pyroxene are characterized as type 7^13^. A-87272 is one of the eucrites that has experienced the strongest impact, with the shock pressure estimated to be ~ 30 GPa^36^. Both pyroxene and plagioclase show a strong undulatory extinction under the cross-polarized light (> 15°). Most of the plagioclase were converted to maskelynite (i.e., shock degree E^3^).

### HaH 262 (type 4, shock degree B)

HaH 262 is an unbrecciated basaltic eucrite, which shows a subophitic texture of coarse pyroxene and lathy plagioclase (Supplementary Fig. 1). The pyroxene in HaH 262 exhibits remnant Ca-zoning with thin augite lamellae (< ~ 5 μm thick) at the rim of host grains (i.e., type 4 eucrites^2^. Both pyroxene and plagioclase show a weak undulatory extinction (< 15°) under cross-polarized light. The plagioclase is partially fractured (i.e., shock degree B^3^).

The datasets used during the current study are available from the corresponding author on reasonable request.

## Supplementary Information

Supplementary Information 1.

Supplementary Information 2.

## Data Availability

The datasets used during the current study are available from the corresponding author on reasonable request.
