# Supplementary material for: Experimental evidence of phase transition of silica polymorphs in basaltic eucrites: implications for thermal history of protoplanetary crust
Source: Sci Rep. 2024 Nov 2;14:26414. doi: 10.1038/s41598-024-77544-x (PMC11531478; doi:10.1038/s41598-024-77544-x)
Supplement: Supplementary file 1 — Supplementary Information 1. [file 41598_2024_77544_MOESM1_ESM.pdf]

Supplementary Figures

Experimental evidence of phase transition of silica polymorphs in basaltic eucrites: Implications for thermal history of protoplanetary crust

Rei Kanemaru, Naoya Imae, Akira Yamaguchi, Aiko Nakato, Junko Isa, Makoto Kimura, Hirotsugu Nishido, Tomohiro Usui, Takashi Mikouchi

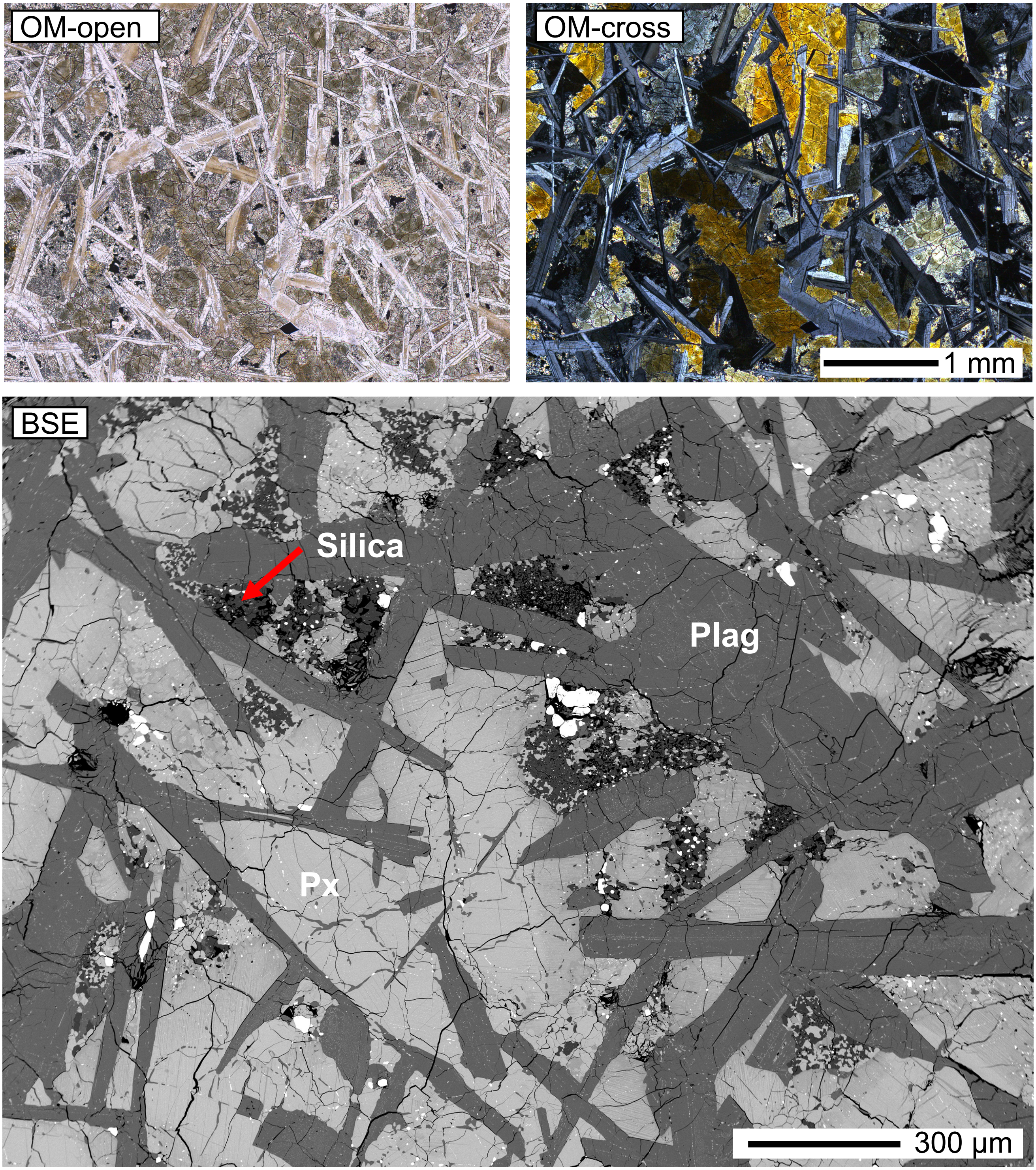

Supplementary Figure 1. Petrographic observation of HaH 262. The pyroxene in HaH 262 shows remnant Ca-zoning with dense thin augite lamellae (i.e., type 4; Takeda and Graham, 1991). Both pyroxene and plagioclase show a weak undulatory extinction (< 15°) under a cross-polarized light (i.e., shock degree B; Kanemaru et al., 2020).

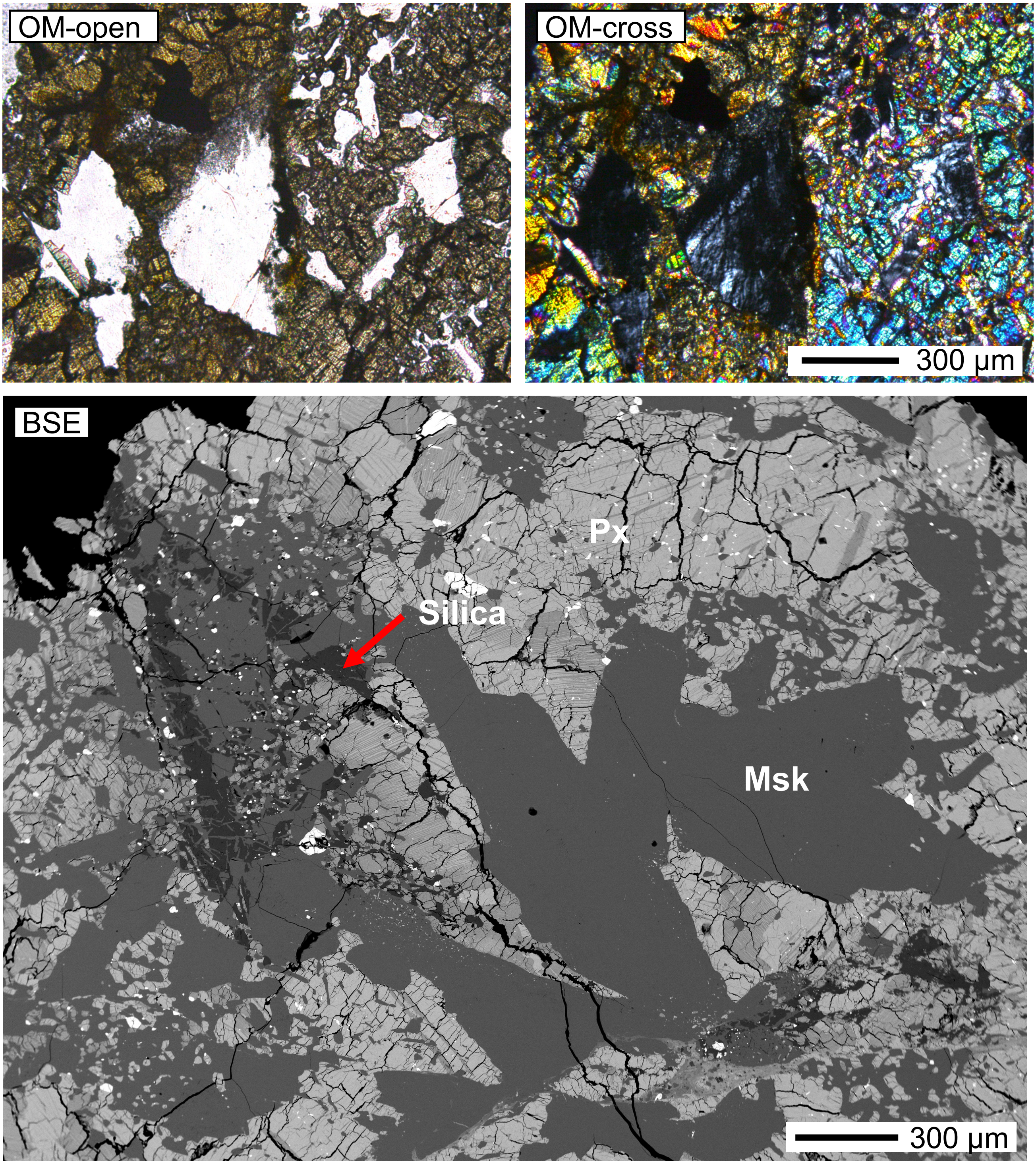

Supplementary Figure 2. Petrographic observation of A-87272. A-87272 is composed of type 7 pyroxene (Yamaguchi et al., 1997) and a large amount of maskelynite (i.e., shock degree E; Kanemaru et al., 2020). The pyroxene shows strong undulatory extinction (>15º) under a cross-polarized light (i.e., shock degree E; Kanemaru et al., 2020).

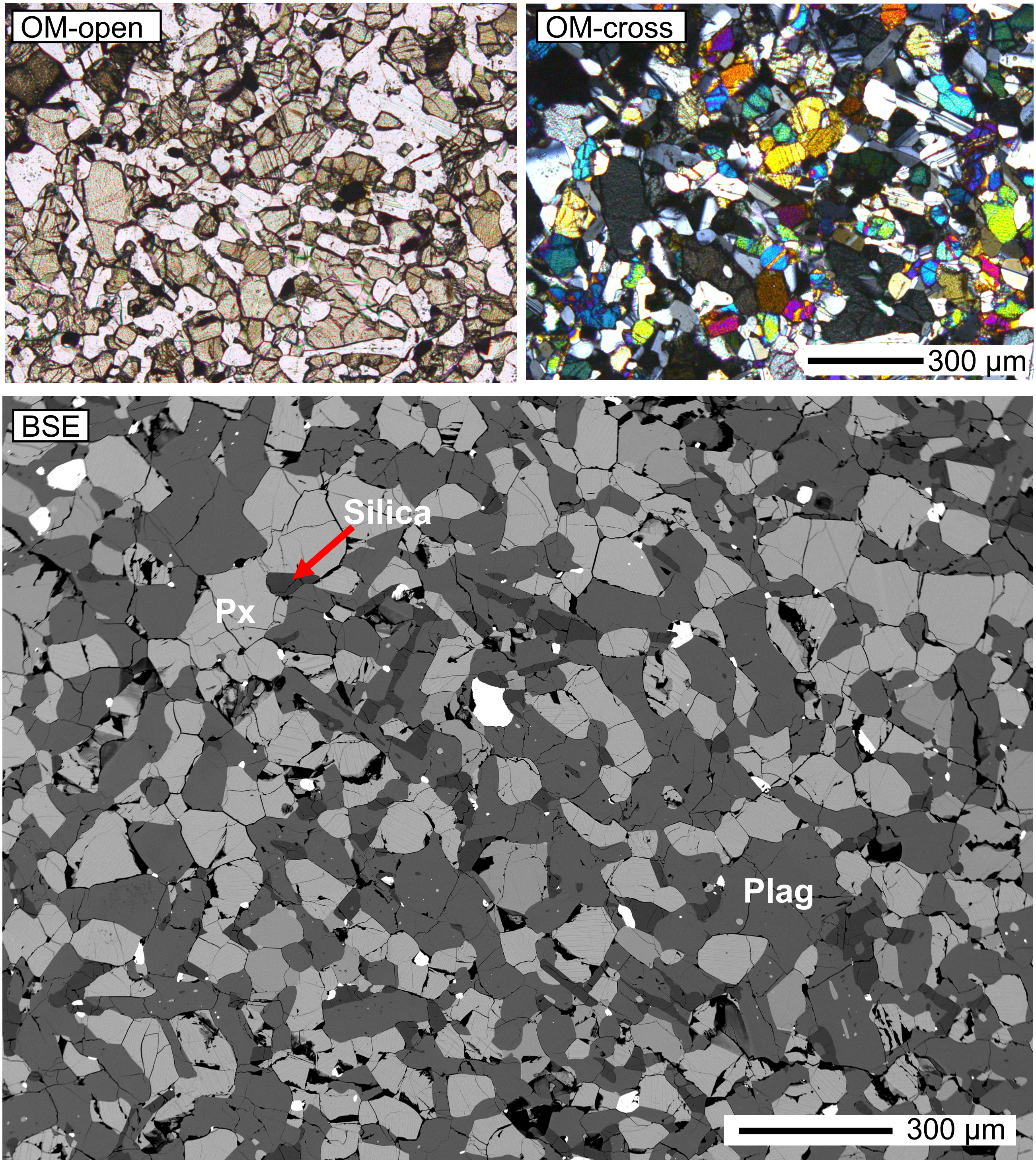

Supplementary Figure 3. Petrographic observation of Agoult. Agoult is composed of fine-grained mineral assemblage (type B recrystallized breccia; Yamaguchi et al., 2009). The pyroxene in Agoult is highly equilibrated, suggesting strong thermal metamorphism (type 5; Takeda and Graham, 1991). Both Pyroxene and plagioclase show a sharp optical extinction (< 2º) under a cross-polarized light (i.e., shock degree A; Kanemaru et al., 2020).

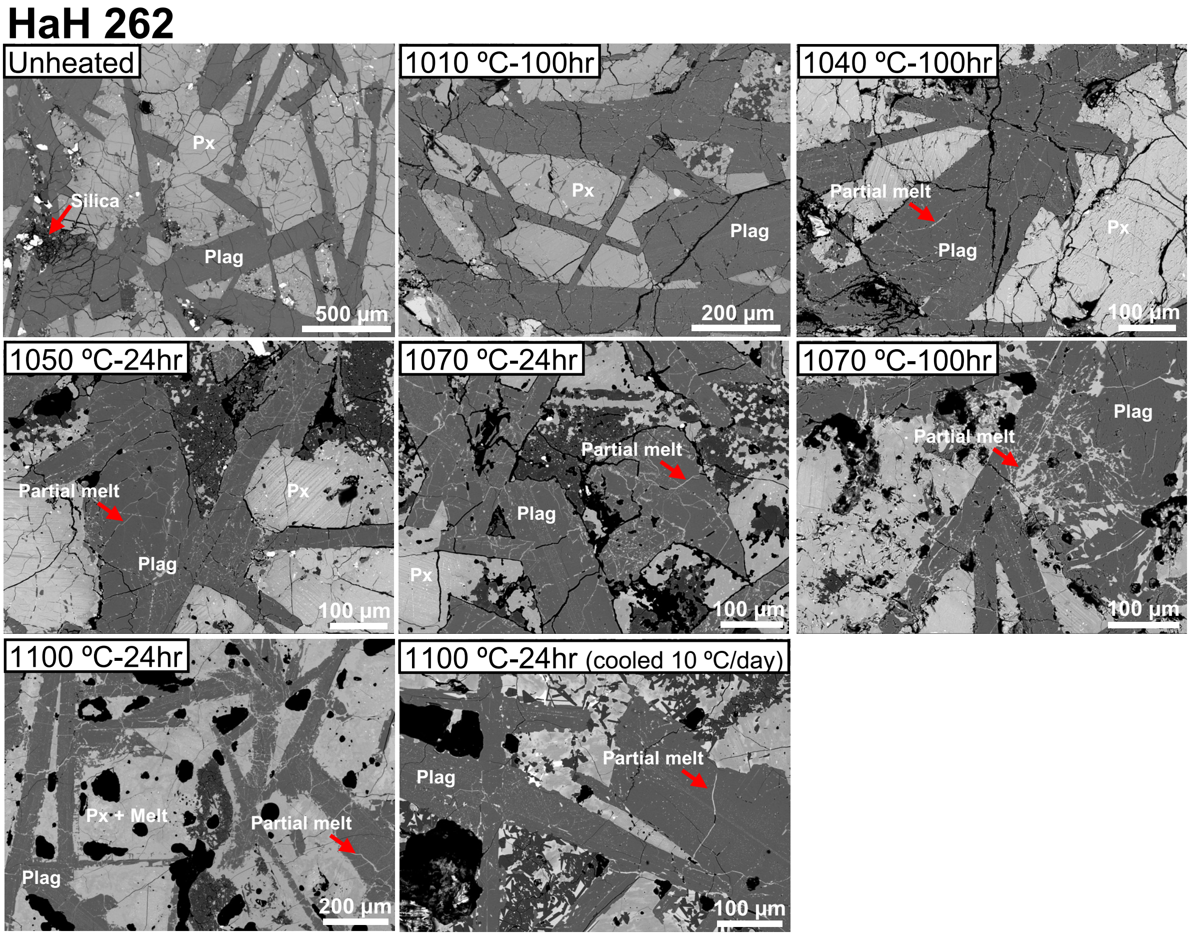

Supplementary Figure 4. BSE images of unheated and experimentally heated HaH 262 from 1010 to 1100 ºC. Shock partial melts are observed in the samples heated at ≥ 1040 ºC.

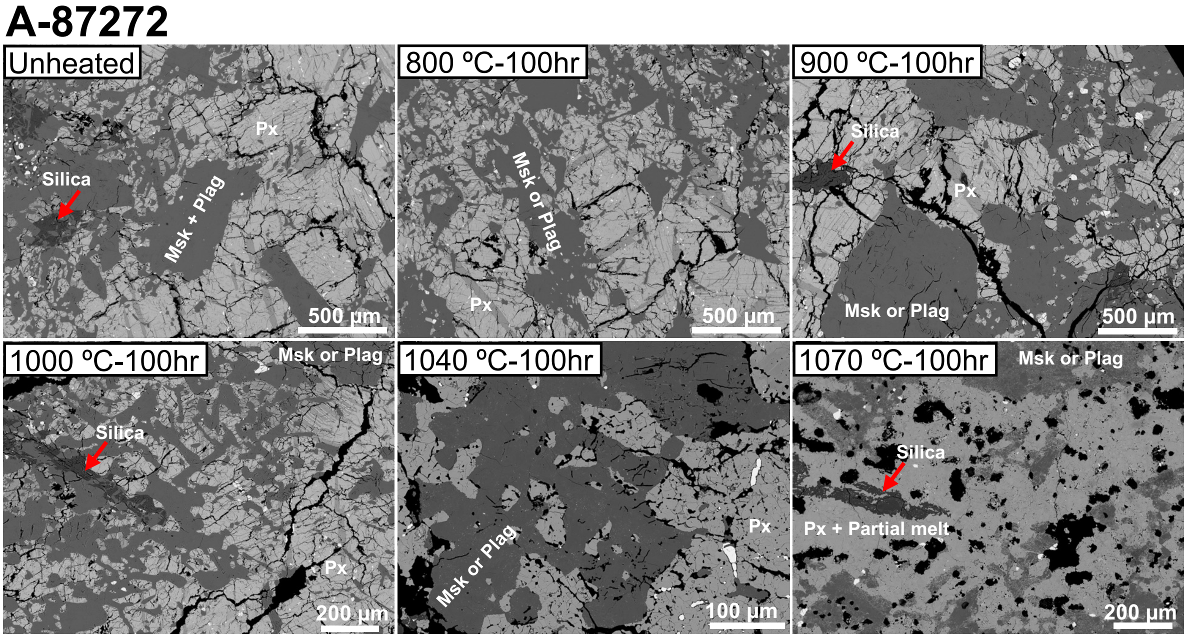

Supplementary Figure 5. BSE images of unheated and experimentally heated A-87272 from 800 to 1070 ºC. Shock partial melts are observed in the samples heated at ≥ 1040 ºC.

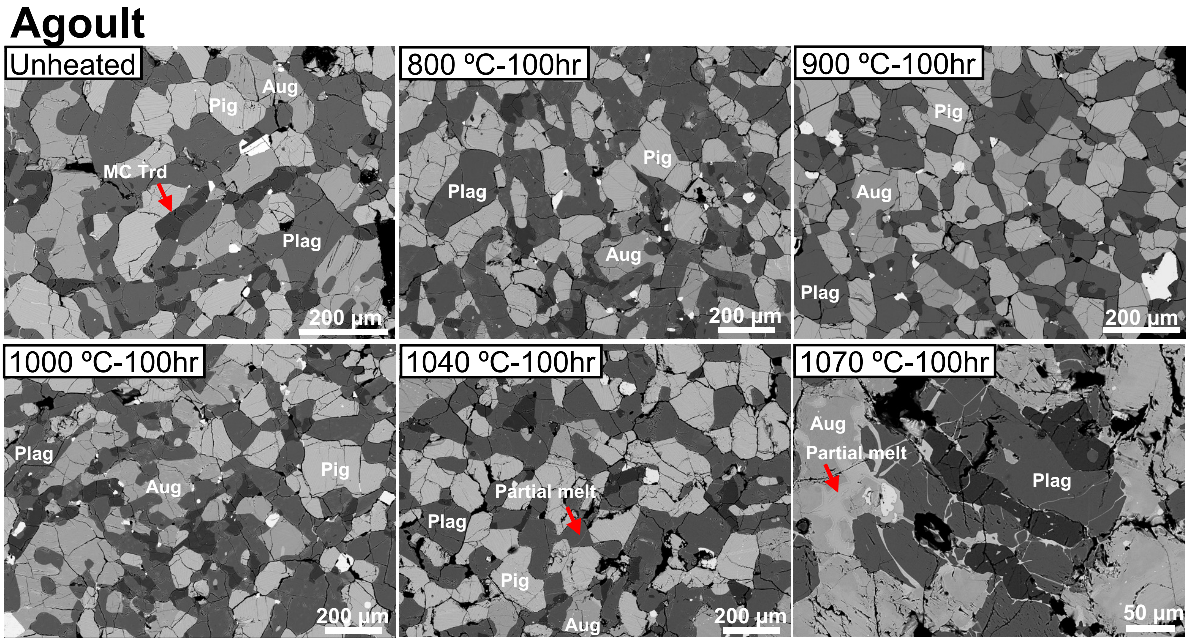

Supplementary Figure 6. BSE images of unheated and experimentally heated Agoult from 800 to 1070 ºC. Shock partial melts are observed in the samples heated at ≥ 1040 ºC.

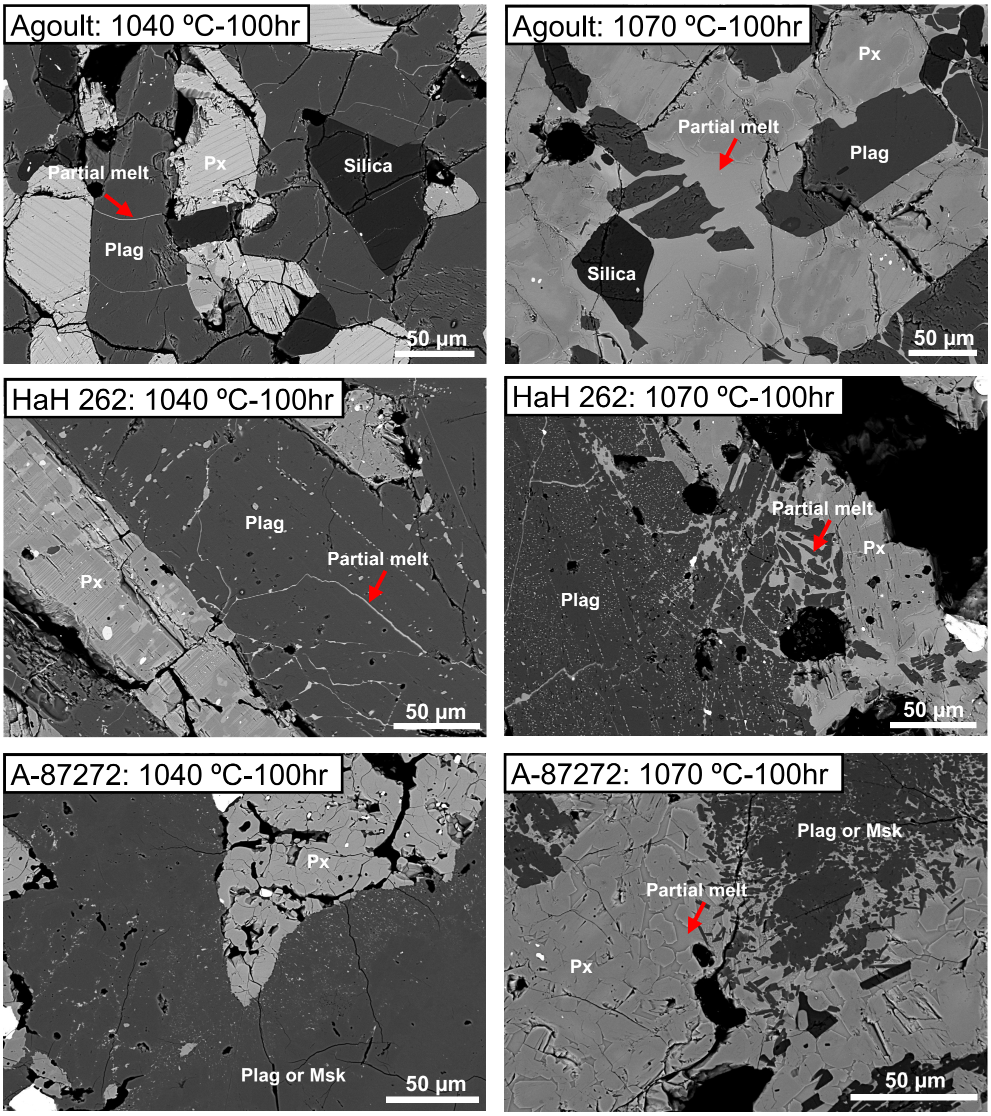

Supplementary Figure 7. BSE images of partial melts in Agoult, HaH 262, and A-87272 experimentally heated at 1040 and 1070 ºC. In the samples heated at 1040ºC, partial melts fill the cracks in the plagioclase. On the other hand, in the samples heated at 1070ºC, the rims portions of the plagioclase become aggregates of fine-grained crystals.

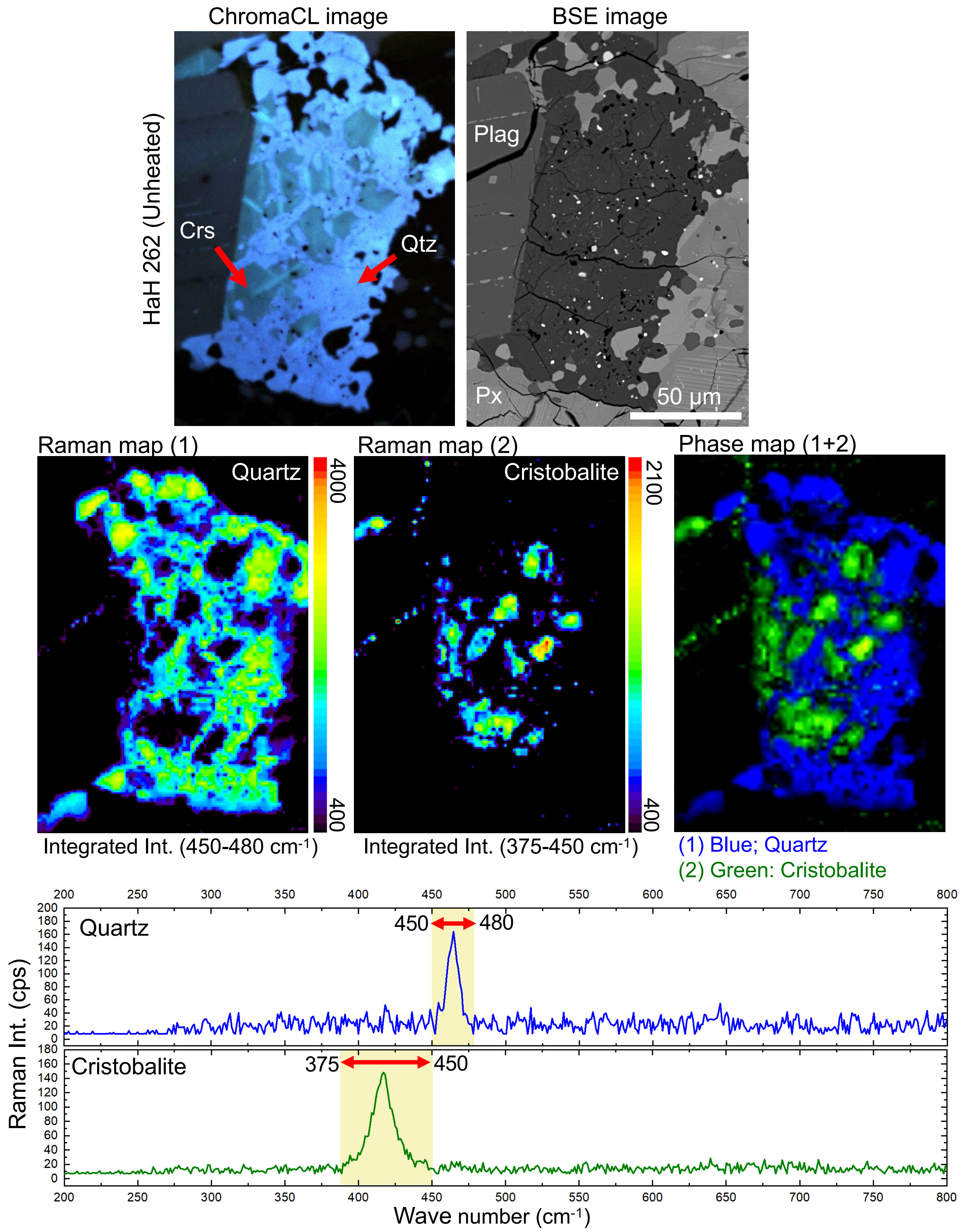

Supplementary Figure 8. Comparison of ChromaCL image and Raman maps. The differences in CL emission in silica grain are represent by Raman mapping to be due to different silica phases. Hence, in this study, we characterized the two-dimensional distribution of silica phases by ChromaCL image and Raman point analyses. The Raman maps were obtained by NRS 5100 at ISAS/JAXA.

Heating experiments 1 (Ex.1) of A-87272

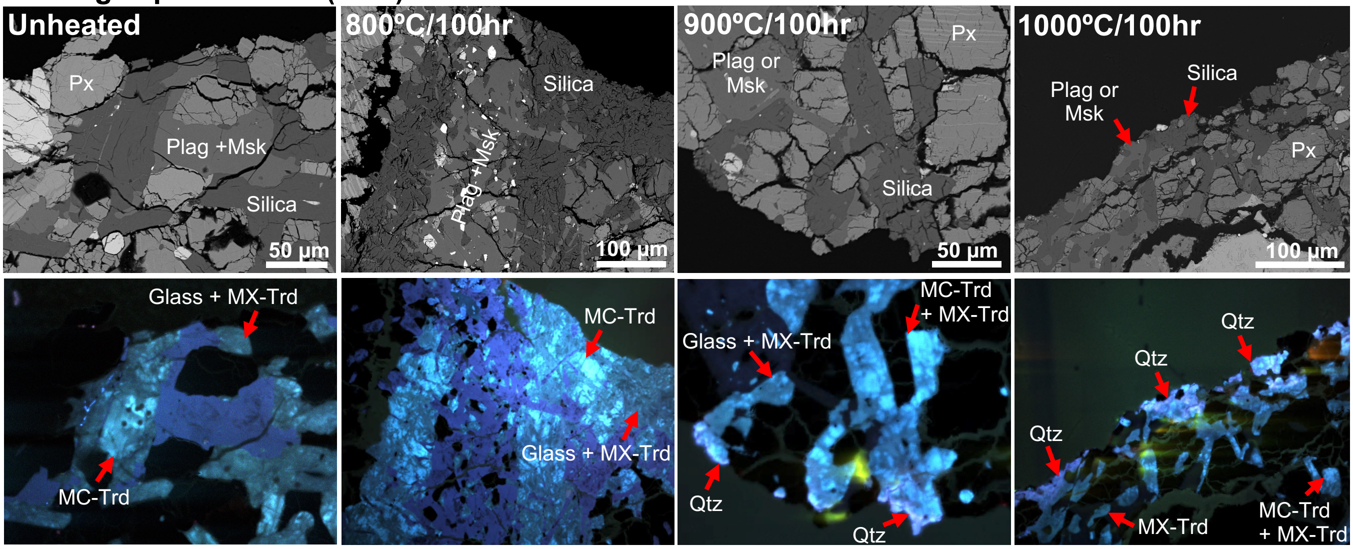

Supplementary Figure 9. BSE and ChromaCL images of silica minerals obtained from the polished sections of unheated and experimentally heated A-87272 (Ex. 1). A purplish CL colors were observed at the edges of the run products heated between 900 and 1000ºC. This texture is absent in samples both unheated and heated at 800ºC. This texture shows the recrystallization of silica glass and MX tridymite into quartz by isothermal heating experiments.

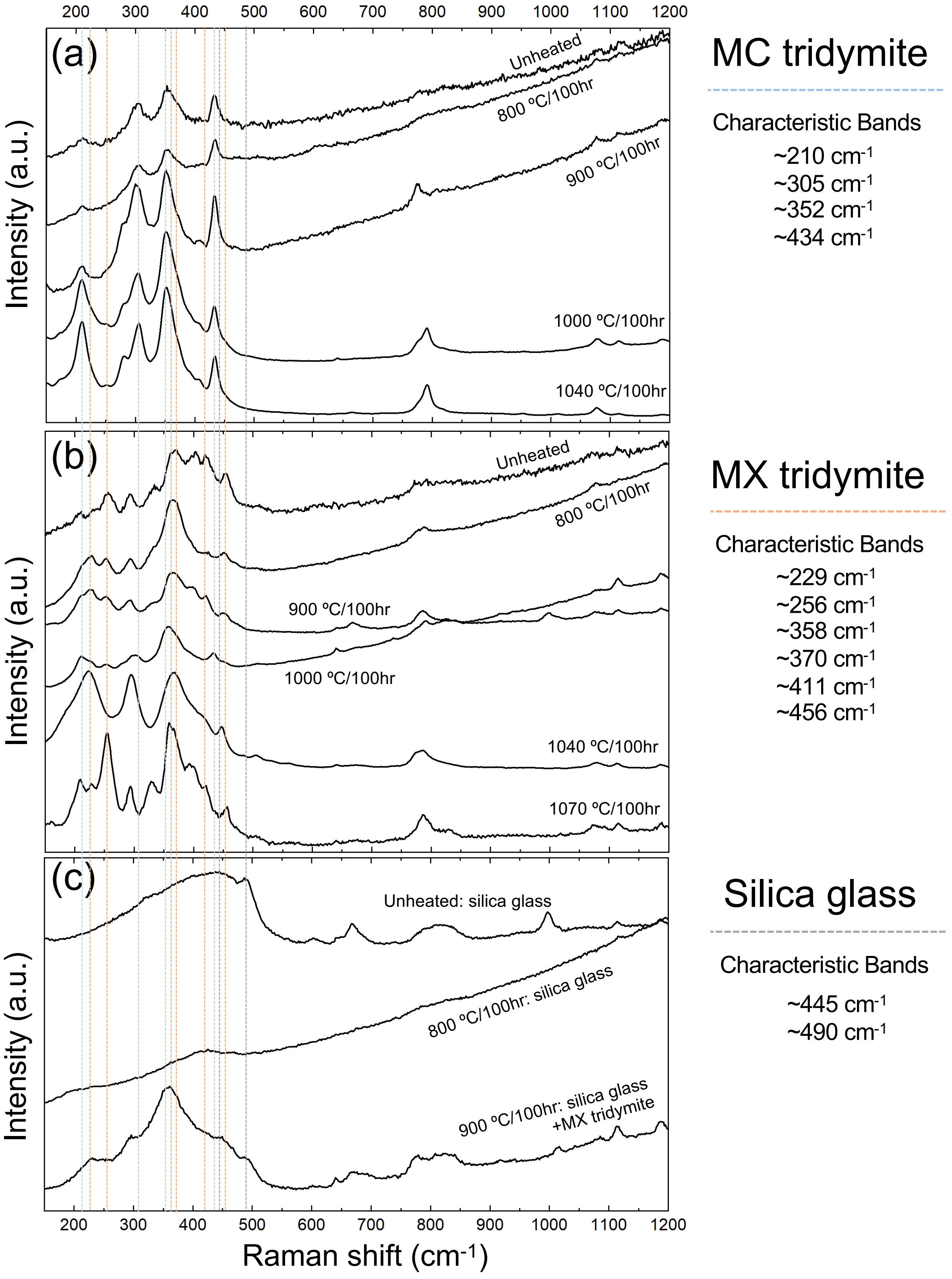

Supplementary Figure 10. Raman spectra of (a) MC tridymite, (b) MX tridymite, and (c) silica glass in unheated and heated A-87272.

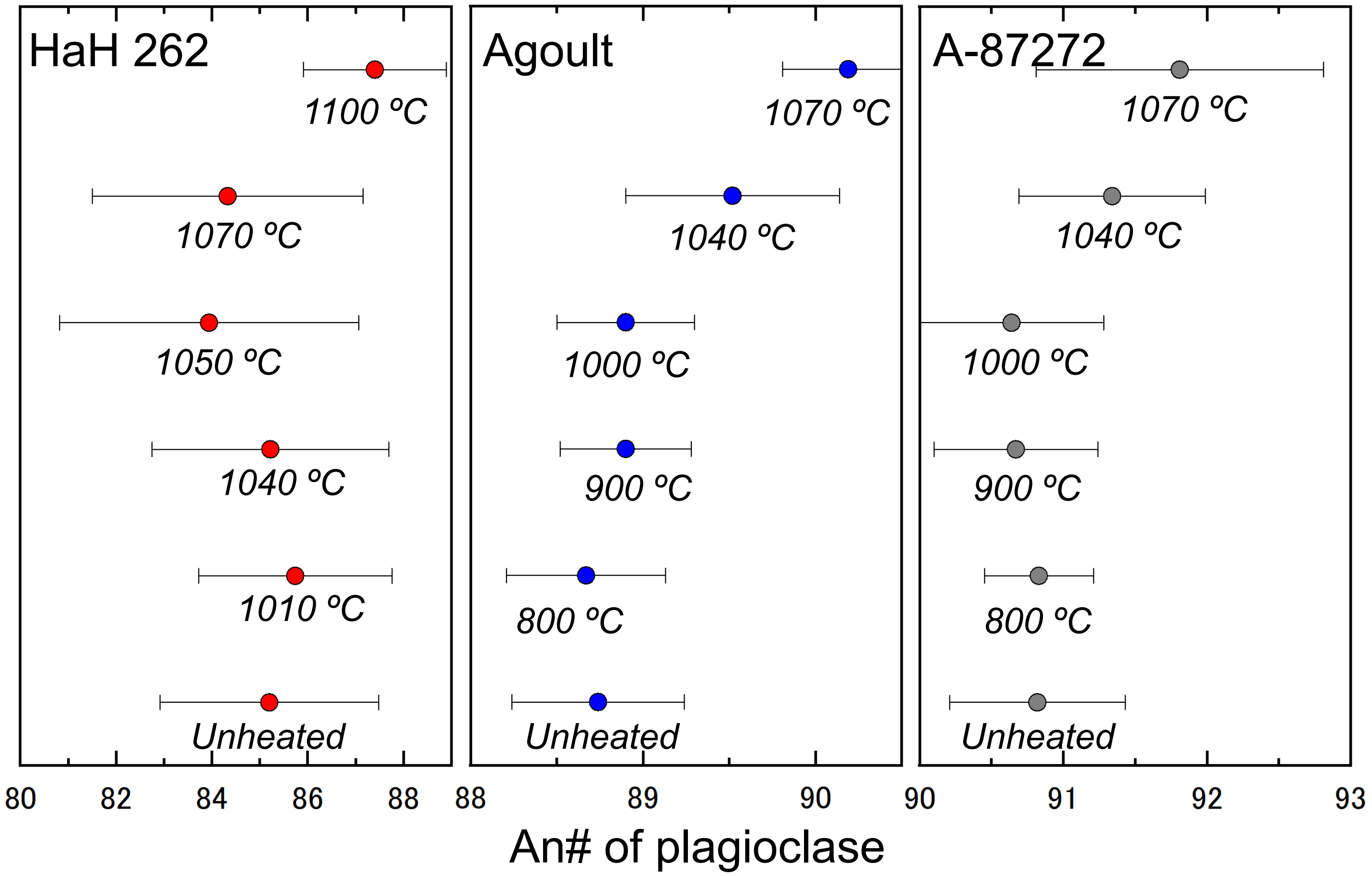

Supplementary Fig. 11. Averaged and standard deviation of An# (Ca/Ca+Na+K cation) with standard deviation in the unheated and heated plagioclase. An increase in the average An# was observed due to heating at temperatures ranging from 1070 to 1100ºC. It suggests that partial melting occurs in the Na-rich rims of plagioclase (Yamaguchi et al., 2013).

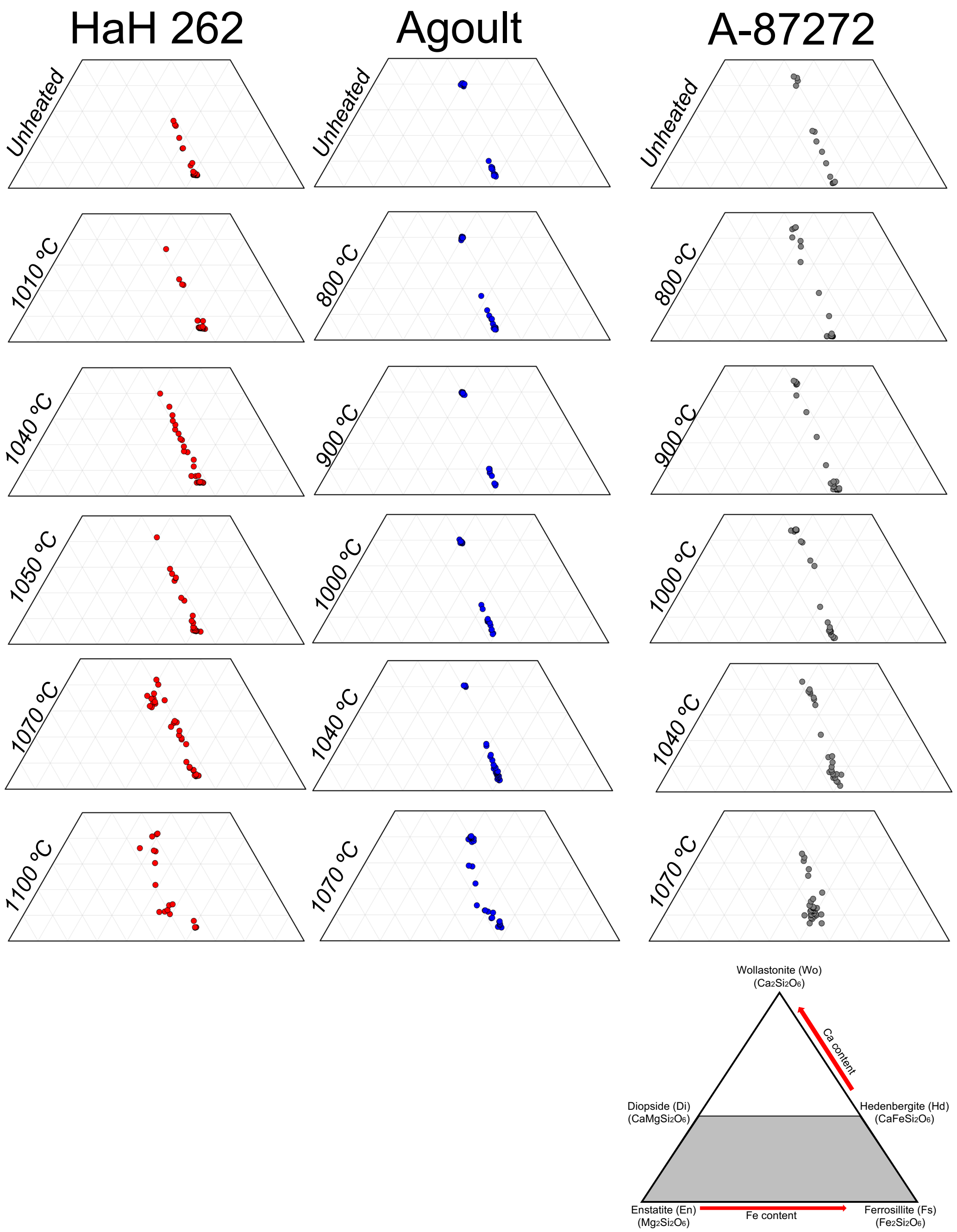

Supplementary Fig. 12. Pyroxene compositions of the studied eucrites. The pyroxene in most heated samples are plotted along a single tie line and remain unchanged from unheated samples. The intermediate Wo contents are due to incomplete resolutions of electron microprobe. Pyroxene in heated above a solidus temperature of eucrite (~1060 ºC), the compositions are scattered toward Mg-rich region. The Mg-rich compositions could be due to the removal of Fe from the melts (Yamaguchi et al., 2013).

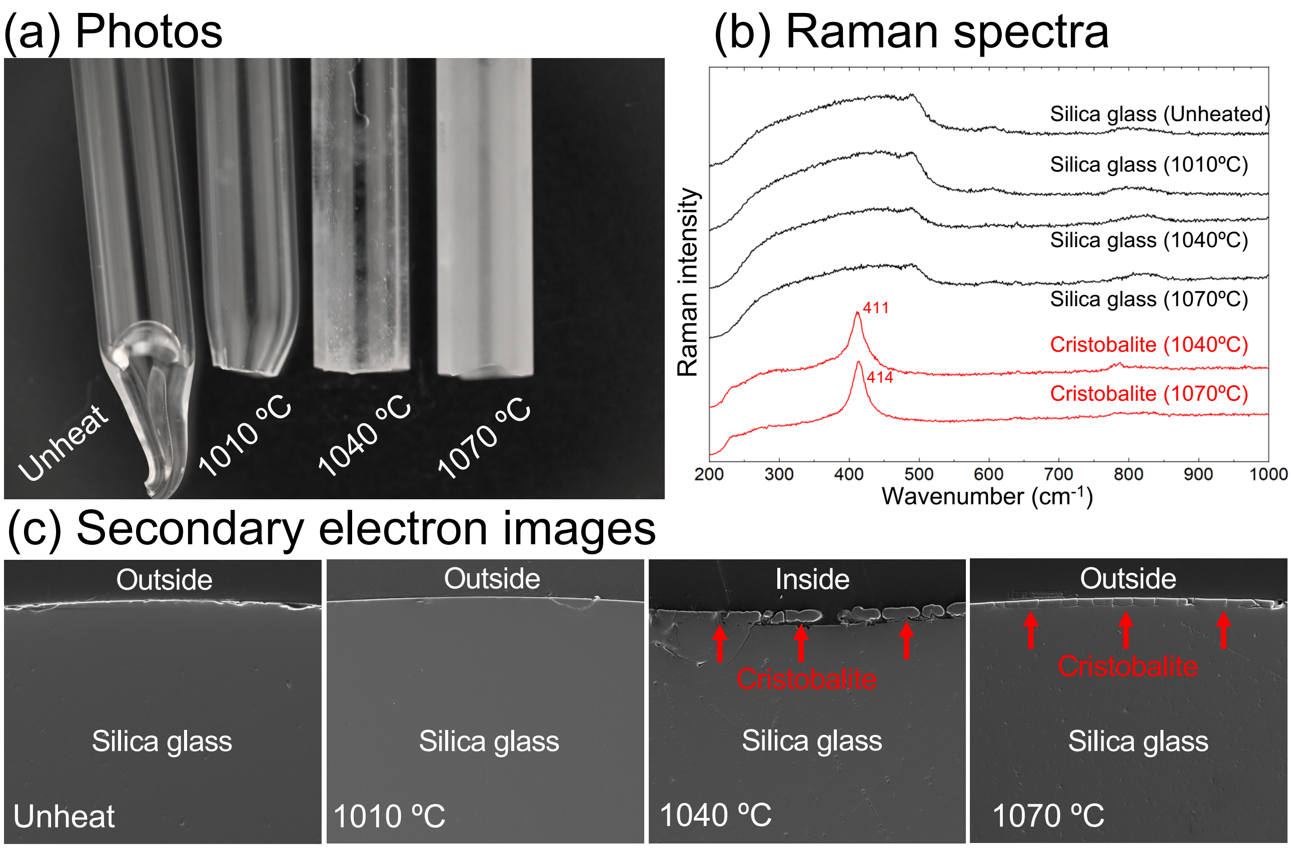

Supplementary Fig. 13. Phase transition of quartz-glass tube in heating experiments at NIPR. (a) photos of unheated and heated quartz-glass tubes. Quartz-glass tubes are transparent both unheated and heated at 1010 ºC. On the other hand, quartz-glass tubes show partial devitrification when heated at 1040 and 1070ºC. (b) Raman spectra of quartz-glass tubes measured by NRS-5100 at ISAS. Raman spectra show that the devitrified portions of the quartz-glass tube are cristobalite, while the transparent portions are silica glass. (c) Secondary electron (SE) images of quartz-glass tubes. Cristobalite is formed on both the inner and outer walls of the quartz-glass tube and contains fine cracks. On the other hand, the interior of the quartz-glass tube remains as silica glass.

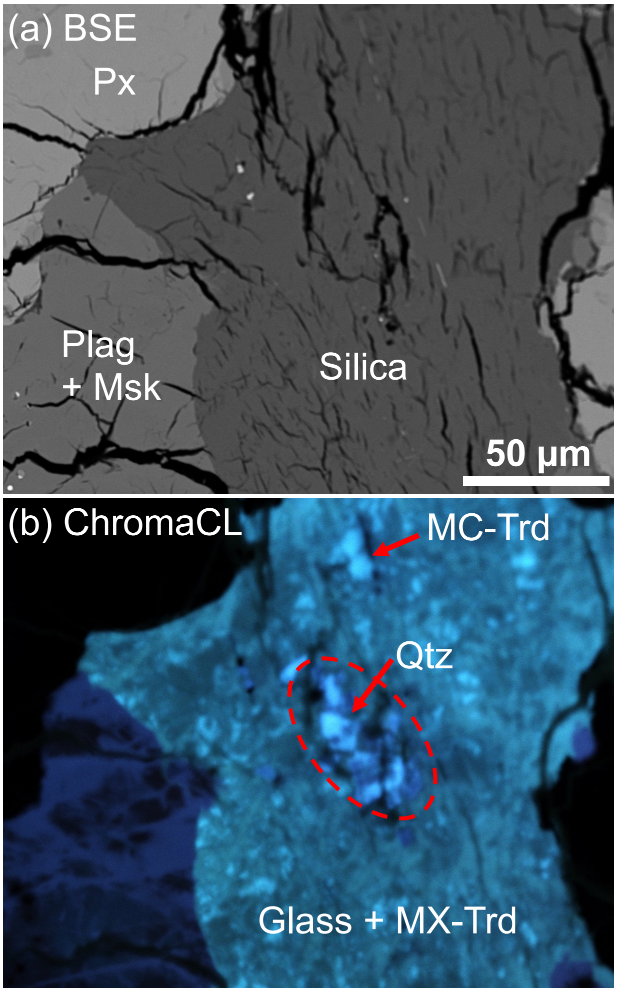

Supplementary Fig. 14. Silica phases in Y 980433, shock degree D cumulate eucrite. (a) BSE and (b) ChromaCL images. The CL color of silica phases is utilized for the identification of silica polymorphs. Quartz and MC tridymite show purplish and bright aqua-blue CL colors, respectively. On the other hand, dark blue CL color portions are corresponding to aggregates composed by silica glass and MX tridymite. Most of silica grains in Y 980433 observed here contain >~50 vol.% of the aggregates composed by silica glass and MX tridymite. Plag = plagioclase; Px = pyroxene; Msk = maskelynite; Qtz = quartz; MC-Trd = monoclinic tridymite, MX-Trd = monoclinic X tridymite.

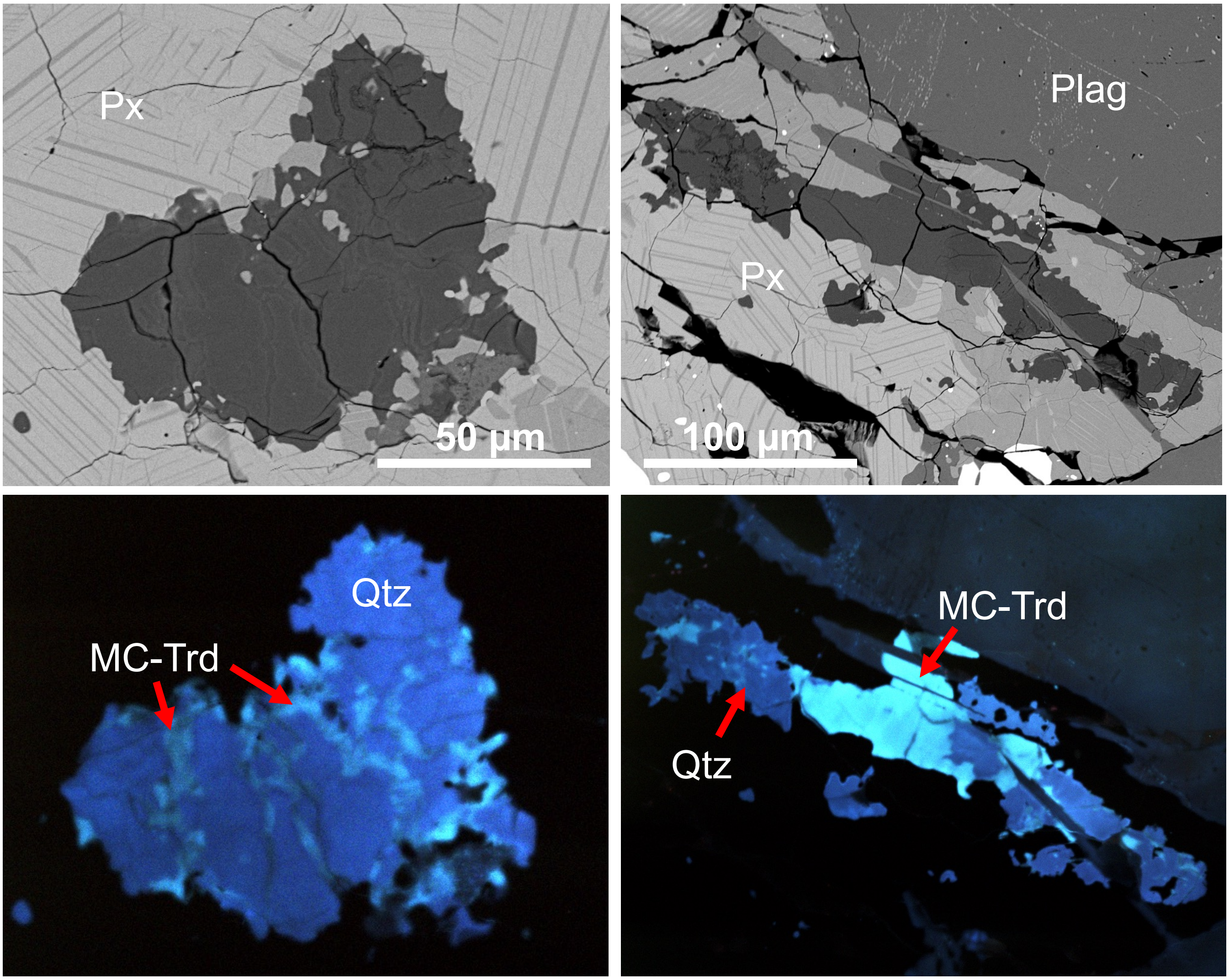

Supplementary Fig. 15. Occurrence of silica minerals in corase grained portion of Juvinas. Quartz and MC tridymite coexist with single a silica particle.
